# Slit2-robo signaling regulates angiogenesis and repair following myocardial infarction

**DOI:** 10.1016/j.yjmcc.2025.10.014

**Published:** 2025-11-05

**Authors:** David Wong, Matthew Tran, Julie Martinez, Itzetl Avila, Adrian Arrieta, Kyle Kalindjian, Elle Rathbun, Thomas M. Vondriska, Eric M. Small, Pearl Quijada

**Affiliations:** a Department of Integrative Biology and Physiology, University of California, Los Angeles, United States; b Molecular, Cellular, and Integrative Physiology Graduate Program, University of California, Los Angeles, United States; c Department of Microbiology, Immunology & Molecular Genetics, University of California, Los Angeles, United States; d Department of Anesthesiology & Perioperative Medicine, David Geffen School of Medicine at the University of California, Los Angeles, United States; e Computational & Systems Biology, University of California, Los Angeles, United States; f Department of Neurology, David Geffen School of Medicine at the University of California, Los Angeles, United States; g Aab Cardiovascular Research Institute, Department of Medicine, University of Rochester School of Medicine and Dentistry, United States; h Eli and Edythe Broad Stem Cell Research Center, University of California, Los Angeles, United States; i Molecular Biology Institute, University of California, Los Angeles, United States

**Keywords:** Myocardial infarction, Vascular biology, Cardiac repair, Robo4, Angiogenesis, Vascular remodeling, Slit2, Robo1, Cardiac fibroblasts, Endothelial cells, AAV9 gene delivery, Hypertrophy

## Abstract

The Slit2 guidance ligand and its Roundabout (Robo) family of receptors regulate axonal guidance and vascular patterning during cardiac morphogenesis, yet the role of Slit2-Robo signaling in the adult heart remains unclear. Here, we identified epicardium-derived Slit2 as highly enriched in neonatal cardiac fibroblasts (cFBs) but markedly reduced in adult hearts. Following myocardial infarction (MI), Slit2 transiently increases in the infarct border zone seven days post-MI but declines significantly after one month. In vitro, Slit2 overexpression in cFBs selectively upregulated angiogenic genes during myofibroblast differentiation without affecting extracellular matrix (ECM) gene expression. In vivo, AAV9-mediated cardiac-specific overexpression of Slit2 (AAV9-cTNT-Slit2) improved cardiac function, increased endothelial cell (EC) proliferation and vascular density, but did not alter fibrotic deposition following MI. Conditioned media from Slit2-overexpressing cFBs promoted EC proliferation, activation, and tube forming abilities, consistent with the increased expression of pro-angiogenic Robo1 and other vascular growth factors in the myocardium of AAV9-Slit2-treated hearts. Additionally, Slit2 overexpression attenuated cardiomyocyte hypertrophy after MI and suppressed fetal gene expression in vitro. Mechanistically, Slit2 appears to mediate its cardioprotective effects through enhanced interactions with Robo1 in cFBs and ECs. These findings support Slit2-Robo signaling as a promising therapeutic target for improving blood vessel formation and maintaining cardiac muscle integrity following ischemic injury.

## 1. Introduction

During cardiac development, the epicardium secretes potent growth factors that guide coronary vasculature formation and promote cardiomyocyte (CM) proliferation and maturation processes essential for morphogenesis [1]. In the adult heart, the epicardium is largely quiescent but continues to produce essential extracellular matrix (ECM) components and signaling molecules that regulate myocardial homeostasis and vascular integrity [2]. After myocardial infarction (MI), the epicardium “re-activates”, upregulating fetal epicardial genes and releasing signaling factors that modulate fibrosis [3], inflammation [4], and angiogenesis [5]. A prevailing hypothesis suggests that effective post-MI repair requires the reactivation of embryonic epicardial gene programs, similar to those observed during cardiogenesis, to mitigate excessive cardiac muscle death and fibrotic infarct expansion [6]. However, single-cell transcriptomics analyses reveal a reduction in pro-regenerative and angiogenic epicardial cell populations in adults compared to developmental stages [7], which may underlie the limited repair capacity of the adult heart following injury. Indeed, harnessing factors from the embryonic or neonatal epicardium has shown potential in promoting endothelial cell (EC) activation and CM proliferation in both in vitro studies and early postnatal models [8,9]. However, the functional benefits of epicardial-derived ligands have not yet been sufficiently validated in adult hearts following injury. Therefore, identifying gene programs within the epicardium that promote vascular growth and maintain myocardial integrity remains a critical gap in reparative cardiac medicine.

Previous studies have revealed that epicardial cells undergoing epithelial-to-mesenchymal transition (EMT) express high levels of axon and vascular guidance genes, which become significantly dysregulated following ablation of EMT [1,10]. Specifically, slit guidance ligand 2 (Slit2)-expressing epicardial-derived cells emerged during EMT and localized near *Robo4*-expressing ECs in the sub-epicardium, positioning Slit2 as a key regulator of coronary vasculature formation [1,11]. Additionally, overexpression of Slit2 in the embryonic epicardium increased venous gene expression in isolated ECs, suggesting that Slit2 may also regulate artery-vein specification [1]. Initially studied for its role in neuronal development, Slit2 is a secreted glycoprotein and a member of a highly conserved family of Slit guidance ligands (SLIT 1–3) that signal through roundabout receptors (ROBO1–4) [12–15]. In the developing mouse heart, Slit2 and Slit3 are expressed in the pericardium, and disruptions in Slit2-Robo signaling have been linked to malformations in the pericardial cavity and caval veins [16,17].

Recent reports indicate SLIT2 is enriched in the embryonic cardiac ECM and neonatal cFBs, and that recombinant SLIT2 promotes CM cytokinesis in early postnatal hearts [18]. Transgenic mice with global overexpression of Slit2 displayed improved cardiac contractility and reduced inflammation following ischemia-reperfusion injury, further implicating Slit2 as a critical factor in preserving CM function during ischemic stress [19]. However, these models do not resolve the specific cell types involved in Slit2-Robo-mediated signaling cross-talk. Moreover, Slit2’s effects on cardiac ECM production remain unclear, although it has been shown to inhibit lung fibrosis by preventing monocyte differentiation into fibrocytes [20]. In contrast, Slit3 exacerbates cardiac fibrosis and myocardial hypertrophy following hypertensive stress, an effect mediated through its interaction with Robo1 expressed in CMs [21]. These findings indicate distinct roles for Slit ligands, with Slit2 showing promising evidence to support the enhancement of cardiac muscle function during ischemic stress.

In the present study, we sought to build on these developmental and injury-related observations. Specifically, we aimed to determine the primary cellular source of Slit2-Robo signaling during postnatal heart growth and disease and investigate whether cardiac-targeted overexpression of Slit2 could improve cardiac repair following MI. Our results demonstrate that Slit2 is enriched in epicardial and fibroblast lineages, with expression declining in the adult heart. Slit2 was acutely induced in cFBs during myofibroblast differentiation in vitro and specifically in the border zone one week after MI. However, its expression was markedly downregulated in chronically infarcted hearts. Adenovirus-mediated overexpression of Slit2 in cFBs did not alter the expression of ECM-associated genes following treatment with transforming growth factor-β1 (TGFβ1) and Angiotensin II (AngII) but instead selectively upregulated the expression of vascular-modifying growth factors. Treatment of the heart with an adeno-associated viral vector serotype 9 (AAV9) containing a cardiac troponin T (cTNT) promoter to drive Slit2 overexpression in the myocardium significantly improved cardiac function up to 28 days post-MI, enhanced vascular cell density, and augmented expression of pro-angiogenic *Robo1*. These findings suggest that Slit2, originating from fibroblasts, plays acritical role in mediating vascular function during ischemia. This is further supported by the observation that conditioned media derived from Slit2-overexpressing cFBs enhance human coronary artery endothelial cell (HCAEC) proliferation, activation, and tube forming capabilities in vitro. Slit2 overexpression also attenuated CM hypertrophy and limited the expression of fetal gene programs associated with cardiac stress in both in vitro and in vivo models. Mechanistically, the cardioprotective effects of Slit2 are likely mediated through activation of Robo1, which is expressed in distinct cardiac cell types, including fibroblasts, ECs, and CMs. These findings provide critical insights into the mechanisms by which Slit2 interacts with various cardiac cell types to drive reparative effects following cardiac injury, underscoring the potential therapeutic implications of Slit2 in facilitating cardiac repair.

## 2. Methods

The Supplemental Material includes all relevant data to this study and a detailed description of the materials and methods. Additional information and data supporting this study are available upon request.

## 3. Results

### 3.1. Slit2 is enriched in epicardial-derived cells and cardiac fibroblasts

We previously showed that *Slit2* is enriched in *Wt1*-lineage epicardial cells that surround *Robo4*^+^ ECs during embryonic development, potentially guiding coronary vasculature formation [1]. After isolation of *Wt1*^+^ epicardial cells, we measured *Slit2* expression from embryonic day (E)12.5 to E16.5 and observed a progressive increase that coincides with EMT, coronary angiogenesis, and vascular maturation (Supplemental Fig. 1A). Fluorescence in situ hybridization (FISH) of E16.5 and 12-week adult hearts revealed significantly higher *Slit2* levels in *Wt1*-lineage cells at E16.5, indicating that Slit2 expression declines as the myocardium reaches maturity (Supplemental Fig. 1B, 1C).

As most epicardial cells differentiate into mesenchymal cells in the heart through EMT [8], we sought to identify the cellular source of Slit-Robo signaling in healthy and diseased adult murine hearts. We analyzed *Slit2* and *Robo* transcript levels in the **C**ardiovascu**LAR A**tlas (CLARA) and CardiacFibroAtlas databases [22–26]. These databases contain single-cell RNA-sequencing datasets of cardiac cells from mice subjected to MI (3- or 7-day post-MI) or AngII infusion (2 weeks) (CLARA), and four independent single-cell RNA sequencing studies of cFB populations enriched in silico from healthy and MI (1-, 3-, 5-, 7-, 14- and 28/30-day post-MI) mouse hearts (CardiacFibroAtlas). In uninjured hearts, *Slit2*, *Slit3,* and *Robo1* were predominantly expressed in cFBs, while *Robo4* was primarily confined to ECs (Supplemental Fig. 2A, 2B). *Slit2, Robo1*, and *Robo4* transcripts were acutely induced after MI, while no significant changes in *Slit2* or *Robo1* were observed following AngII treatment (Supplemental Fig. 2C, 2D), suggesting that increased Slit2 and its corresponding receptors are molecular hallmarks of ischemia. Analysis of the integrated fibroblast-specific dataset revealed that *Slit2* transcripts were enriched in injury-associated fibroblast subtypes: Matrifibrocytes (MFC), Myofibroblasts (MYO), Injury Response (IR), and Cycling (F–Cl) (Supplemental Fig. 3A). *Robo1* exhibited a similar expression pattern post-MI, with additional expression in Wnt-expressing (F-Wntx) fibroblasts, a subtype previously implicated in tissue remodeling and repair [24] (Supplemental Fig. 3B). *Slit2* and *Robo1* expression peaked around 7 days post-MI, suggesting that Slit2-Robo signaling is most active during the acute stages of ischemia (Supplemental Fig. 3A, B). Based on these findings, we focused our investigation on evaluating the endogenous expression of Slit2 and its receptor *Robo1* across cardiac development, homeostasis, and post-ischemic remodeling.

**Fig. 1.**
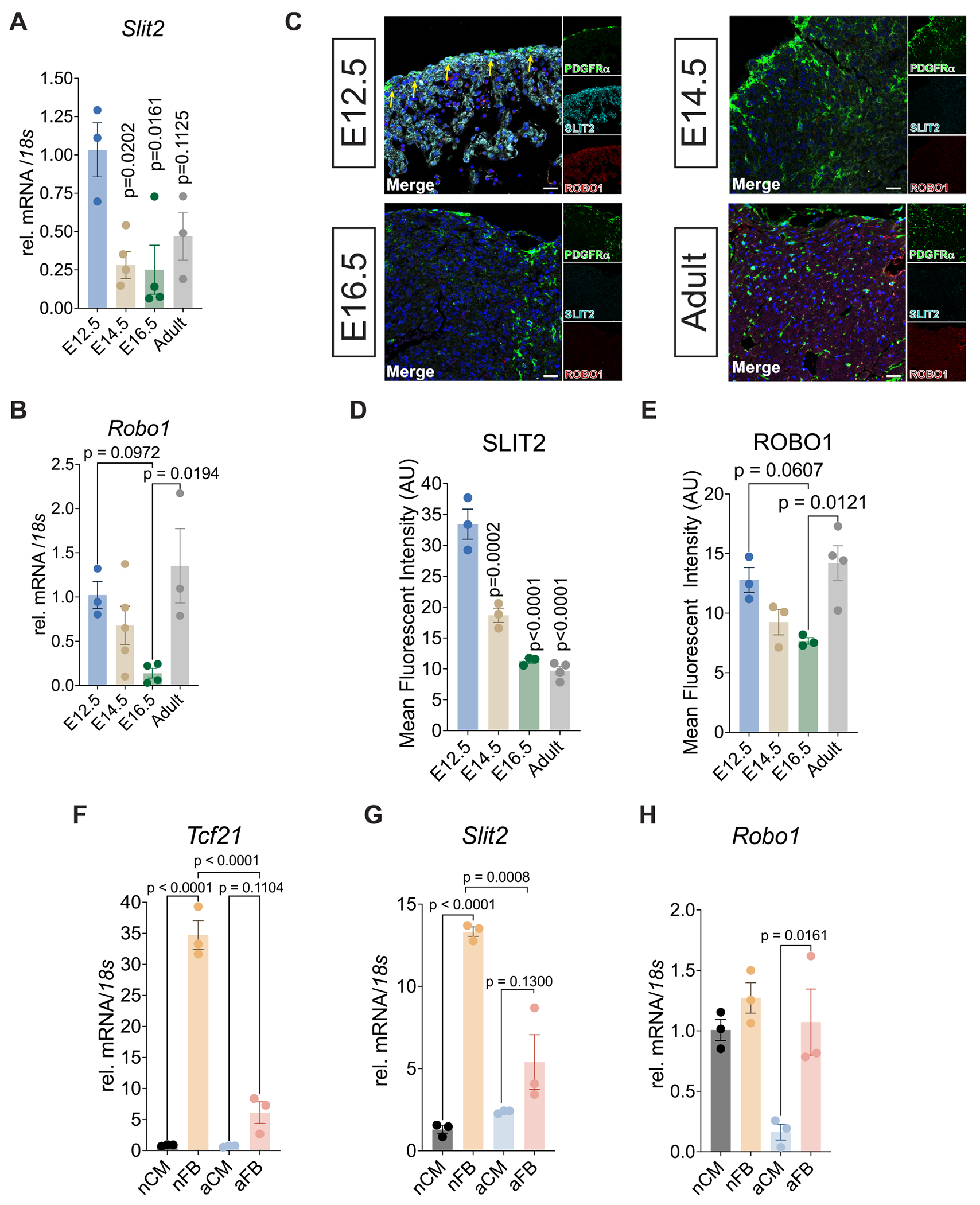
Slit2 is enriched in cardiac fibroblasts. A, Relative expression of *Slit2* and B, *Robo1* in whole heart tissue isolated from embryonic day (E) 12.5, E14.5, E16.5, and 12-week-old adult mice. *N* = 3 per sample. *P* value determined by One-Way ANOVA. C, Heart sections from E12.5, E14.5, E16.5, and adult mice. Representative images of sections labeled with antibodies against SLIT2 (Cyan), ROBO1 (red), and PDGFRα (Green). DAPI staining was used to visualize nuclei (blue). Yellow arrows highlight SLIT2^+^ cells. Micrographs represent N = 3 biological samples imaged at each time point. Scale bar, 20 μm. D, Mean fluorescence intensity of SLIT2 and E, ROBO1. N = 3 for embryonic hearts and *N* = 4 for adult hearts. Gene expression of *Tcf21* (F), *Slit2* (G), and *Robo1* (H) in cardiomyocytes (CM) and fibroblasts (FB) isolated from neonatal (n) and 12-week-old adult hearts (a). N = 3 per sample. P value determined by One-Way ANOVA. Data were presented as mean +/− standard error of the mean (SEM). (For interpretation of the references to colour in this figure legend, the reader is referred to the web version of this article.)

Next, we assessed the expression patterns of Slit2 and Robo1 transcripts and protein levels in the whole heart. Interestingly, *Slit2* levels in whole cardiac tissue differed from those in epicardial cells, showing peak expression at E12.5 and gradually decreasing through E14.5, E16.5, and into adulthood (Fig. 1A). In contrast, *Robo1*, while reduced in the later stages of embryonic development, exhibited increased expression in adult hearts (Fig. 1B). SLIT2 and ROBO1 protein levels mirrored their transcriptional trends at all time points (Fig. 1C–E). To further define the cellular expression of *Slit2* and *Robo1*, we isolated CMs and cFBs from postnatal day 1 (P1) and adult mice and performed gene expression analysis. The fibroblast gene marker *Tcf21* was enriched in cFBs compared to CMs in both age groups (Fig. 1F). *Slit2* expression was markedly higher in P1 cFBs compared to P1 CMs, but decreased significantly in adult-derived cFBs (Fig. 1G). In contrast, *Robo1* transcript levels were similar between neonatal cFBs and CMs, but became restricted to cFBs in the adult heart (Fig. 1H). These results suggest that Slit2 is primarily expressed in epicardial-derived cells and cFBs, with peak expression occurring during embryogenesis and declining in adulthood. Robo1, while broadly expressed in the embryonic heart, becomes fibroblast-specific in adulthood, indicating a developmental shift in receptor localization.

### 3.2. Slit2 is transiently upregulated during acute cardiac stress

**Fig. 2.**
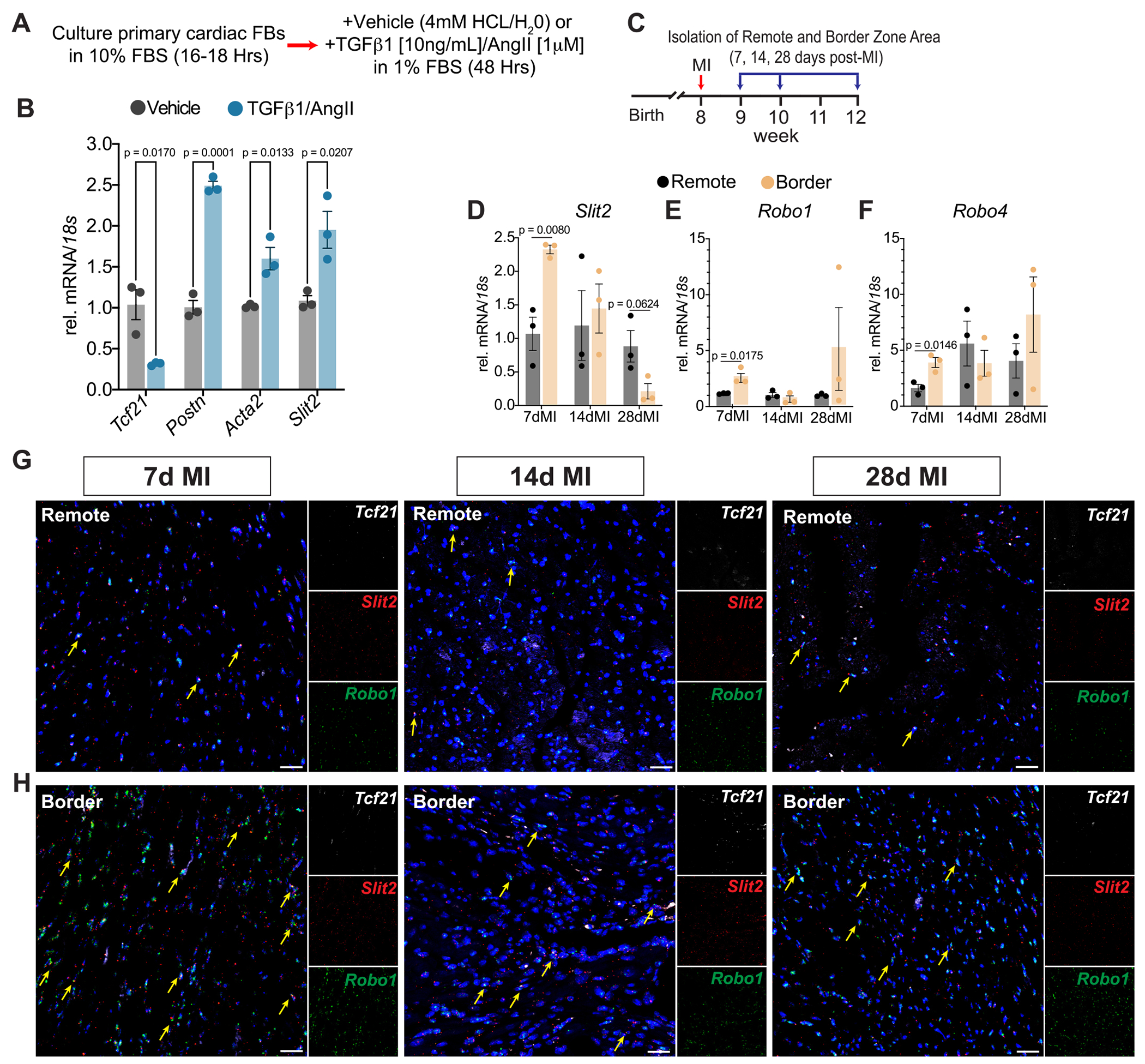
Slit2 is transiently induced during acute cardiac stress. A, Schematic of experimental strategy for activating primary cardiac fibroblasts in vitro. B, *Tcf21, Postn, Acta2,* and *Slit2* in primary cardiac fibroblasts. *N* = 3. *P* values were determined by a Student’s *t*-test between Vehicle and TGFβ1/AngII treatment for each gene. C, Schematic of the experimental timeline of permanent ligation of the left anterior artery to induce myocardial infarction on *C57BL/6 J* mice before isolation of the remote and border zone regions of the left ventricle. D, *Slit2,* E, *Robo1,* and F, *Robo4* in the border zone tissue compared to the remote zone tissue at 7-, 14-, and 28-days post-myocardial infarction. N = 3 per region/time-point. P values were determined by a Student’s *t*-test between the Remote and Border regions at each time-point. RNA-FISH was used to demonstrate expression of *Slit2*, *Tcf21*, and *Robo1* in the remote (G) and border zone regions (H) of the heart at 7-, 14-, and 28-day post-MI. DAPI staining was used to visualize nuclei (blue). Yellow arrows, Slit2^+^ Robo1^+^ cells. Micrographs represent N = 3 biological samples imaged at each time point. Scale bar, 20 μm. Data were presented as mean +/− standard error of the mean (SEM). (For interpretation of the references to colour in this figure legend, the reader is referred to the web version of this article.)

To assess whether *Slit2* is altered during fibroblast activation, we treated primary cFBs with pro-fibrotic stimuli, TGFβ1 and AngII, for 48 h (Fig. 2A). AngII enhances TGFβ1 signaling, reinforcing the phenotypic transition of FBs to myofibroblasts [27,28]. Following TGFβ1/AngII treatment, fibroblast activation was confirmed by the downregulation of the quiescent fibroblast gene marker *Tcf21*, along with the upregulation of myofibroblast genes *Postn* and *Acta2* (Fig. 2B). Notably, *Slit2* expression significantly increased in cFBs after 48 h of TGFβ1/AngII stimulation (Fig. 2B), consistent with our transcriptional analysis showing Slit2 induction in cFBs 7 days post-MI (Supplementary Figs. 2 and 3).

Following MI, a distinct border zone (BZ) forms between the injured, hypoxic tissue and the surviving, normoxic myocardium, characterized by variable mechanical stress, a heterogeneous cellular composition, and differential gene expression compared to uninjured remote zones (RZ) [29–31]. Given our prior findings that Slit2 is essential for developmental cardiac remodeling [1] and is upregulated in activated cFBs (Fig. 2B), we hypothesized that Slit2 may be elevated in the BZ after MI to support vascular growth. To investigate this, we induced MI in *C57BL/6 J* mice via permanent ligation of the left anterior descending artery [32] and micro-dissected myocardial BZ and RZ tissue at 7, 14, and 28 days post-MI to conduct gene expression analysis (Fig. 2C). *Slit2* was significantly elevated in the BZ at 7 days post-MI but declined by day 28, falling below levels observed in the RZ (Fig. 2D). *Robo1* and *Robo4* were transiently upregulated at 7 days post-MI, with sustained expression at later time points (Fig. 2E, F). These dynamic expression patterns align with the observed increases in *Slit2*, *Robo1*, and *Robo4* in transcriptomic data from ischemic hearts (Supplementary Figs. 2). FISH further confirmed *Slit2* and *Robo1* induction in the BZ, with *Slit2* co-localizing with *Robo1*^+^ cells at 7 days post-MI compared to the remote region (Fig. 2G, H). Collectively, these data indicate that Slit2 is upre- gulated in cFBs in response to TGFβ1/AngII stimulation in vitro and during acute ischemic injury, where it co-localizes with *Robo1*-expressing cells in the injured myocardium.

### 3.3. Overexpression of Slit2 in cardiac fibroblasts promotes the expression of angiogenic vascular genes

**Fig. 3.**
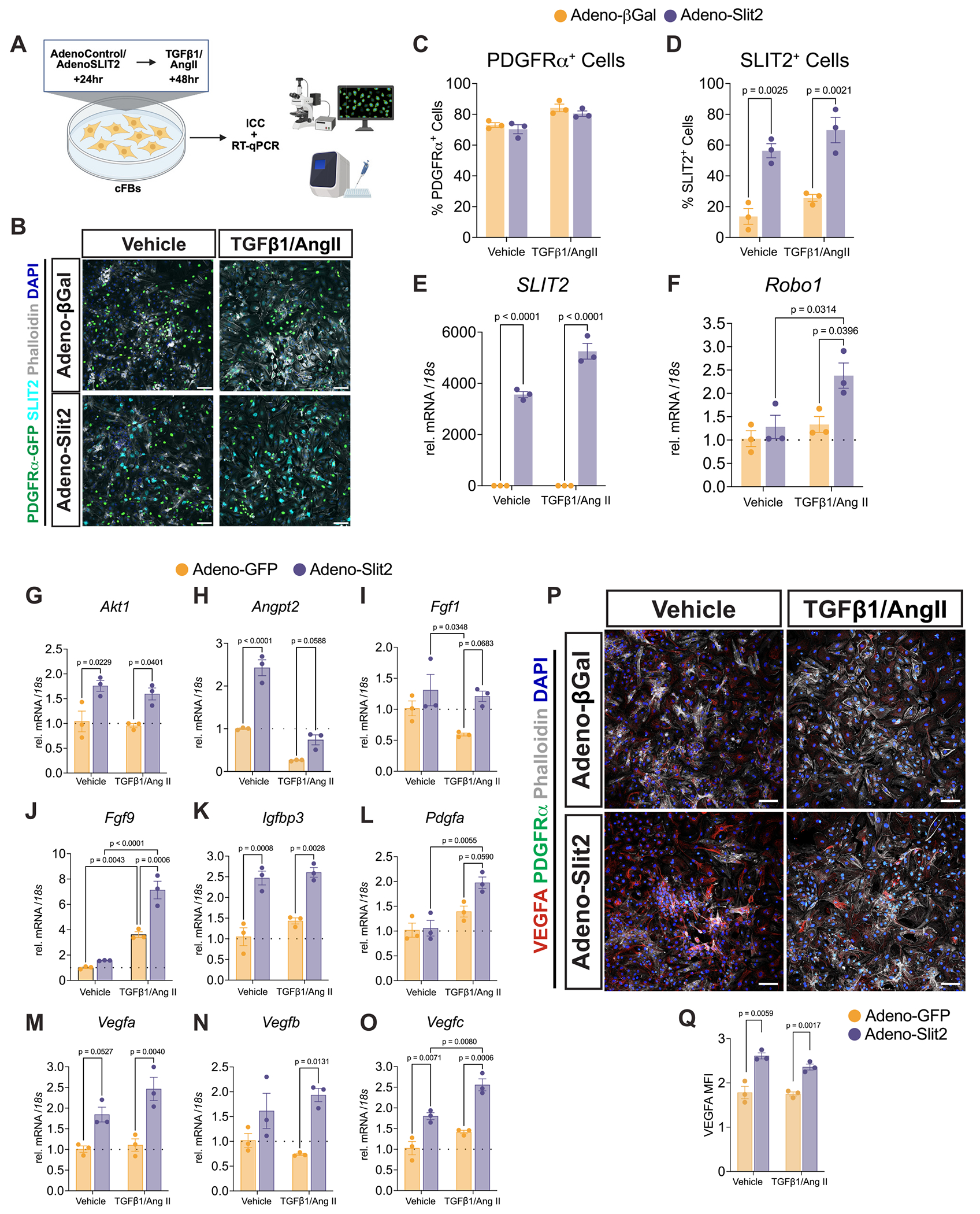
Overexpression of Slit2 in cardiac fibroblasts promotes the expression of angiogenic vascular genes A, Schematic of experimental design: primary cardiac fibroblasts (cFBs) isolated from postnatal day 1 (P1) mice were infected with adenovirus expressing GFP or βgal (AdenoControl) or human SLIT2 (AdenoSLIT2) for 24 h, followed by treatment with TGFβ1/AngII or Vehicle for 48 h. Cells were harvested for RT-qPCR and immunocytochemistry analyses. B, Representative immunofluorescence images of cFBs isolated from *PDGFRα*^*nGFP/*+^ reporter mice treated with adenoviruses and either vehicle or TGFβ1/AngII. Cells were labeled with antibodies against GFP (PDGFRα, green), SLIT2 (cyan), Phalloidin (F-Actin, gray), and DAPI (blue). Scale bar, 20 μm. C, Percentage of PDGFRα^+^/DAPI^+^ cells and D, SLIT2^+^/DAPI^+^ cells per field across conditions. *N* = 3 per condition. Expression of *SLIT2* (E) and *Robo1* (F), or vascular-related genes (G-O) following vehicle or TGFβ1/AngII treatment in AdenoGFP or AdenoSLIT2-treated cFBs. *N* = 3. Statistical comparisons were made using two-way ANOVA with Tukey’s multiple comparisons test to determine *P* values. (P-Q) Immunocytochemistry analysis of VEGFA protein expression in cFBs infected with AdenoBGal or AdenoSLIT2 and treated with vehicle or TGFβ1/AngII. Representative immunofluorescence images (P) show VEGFA (red), Phalloidin (F-Actin, gray), and DAPI (blue). Scale bar, 20 μm. Quantification of VEGFA mean fluorescence intensity (MFI) in (Q). N = 3. Statistical comparisons were made using an unpaired student’s *t*-test between conditions. Data were presented as mean +/− standard error of the mean (SEM). (For interpretation of the references to colour in this figure legend, the reader is referred to the web version of this article.)

To assess whether Slit2 overexpression alters fibroblast activation, primary neonatal cFBs were isolated from *PDGFRα*^*nGFP/*+^ [33] and *C57BL/6 J* mice, followed by transduction with adenoviruses expressing either human SLIT2 (Adeno-Slit2) or control viruses to express β-galactosidase (Adeno-βGal) or green fluorescent protein (Adeno-GFP). Cells were treated with TGFβ1/AngII to induce myofibroblast differentiation (Fig. 3A). Cultured primary cFBs were shown to be up to 70 % PDGFRα^+^ based on immunocytochemical analysis of nuclear GFP (Fig. 3B, C). Slit2 protein and mRNA levels were robustly elevated in Adeno-Slit2-treated cells compared to Adeno-βGal/GFP-treated controls (Fig. 3B, D, E). Interestingly, *Robo1* was significantly upregulated in Adeno-Slit2-treated cFBs, but only following TGFβ1/AngII treatment (Fig. 3F). As expected, TGFβ1/AngII treatment led to downregulation of *Tcf21* and upregulation of ECM-related genes such as *Col1a1, Col3a1, Col4a1, Col5a1, Acta2, Fn,* and *Postn* compared to vehicle-treated cells (Supplemental Fig. 4A–H). However, SLIT2 overexpression did not significantly alter the expression of ECM-related genes relative to control cells after TGFβ1/AngII treatment (Supplemental Fig. 4A–H), indicating that SLIT2 does not alter fibrotic gene programs under activation conditions in vitro. Instead, *SLIT2* overexpression resulted in increased expression of vascular-associated signaling genes, including *Akt1*, *Angpt2*, *Fgf1*, *Fgf9*, *Igfpb3*, *Pdgfa*, *Vegfa*, *Vegfb,* and *Vegfc,* observed both with and without TGFβ1/AngII stimulation (Fig. 3G–O). Notably, the upregulation of *Akt1* coincided with increased *Robo1* expression, suggesting activation of pro-angiogenic VEGFR2-associated signaling pathways [34]. VEGFA and FGF9 protein in Slit2-overexpressing cFBs were increased relative to controls, consistent with gene expression data (Fig. 3P, Q, and Supplemental Fig. 4I, 4J). Altogether, these findings suggest that Slit2 enhances the angiogenic potential of cFBs by promoting the expression of vascular growth factors in vitro.

### 3.4. Slit2 enhances cardiac functional recovery following myocardial infarction

**Fig. 4.**
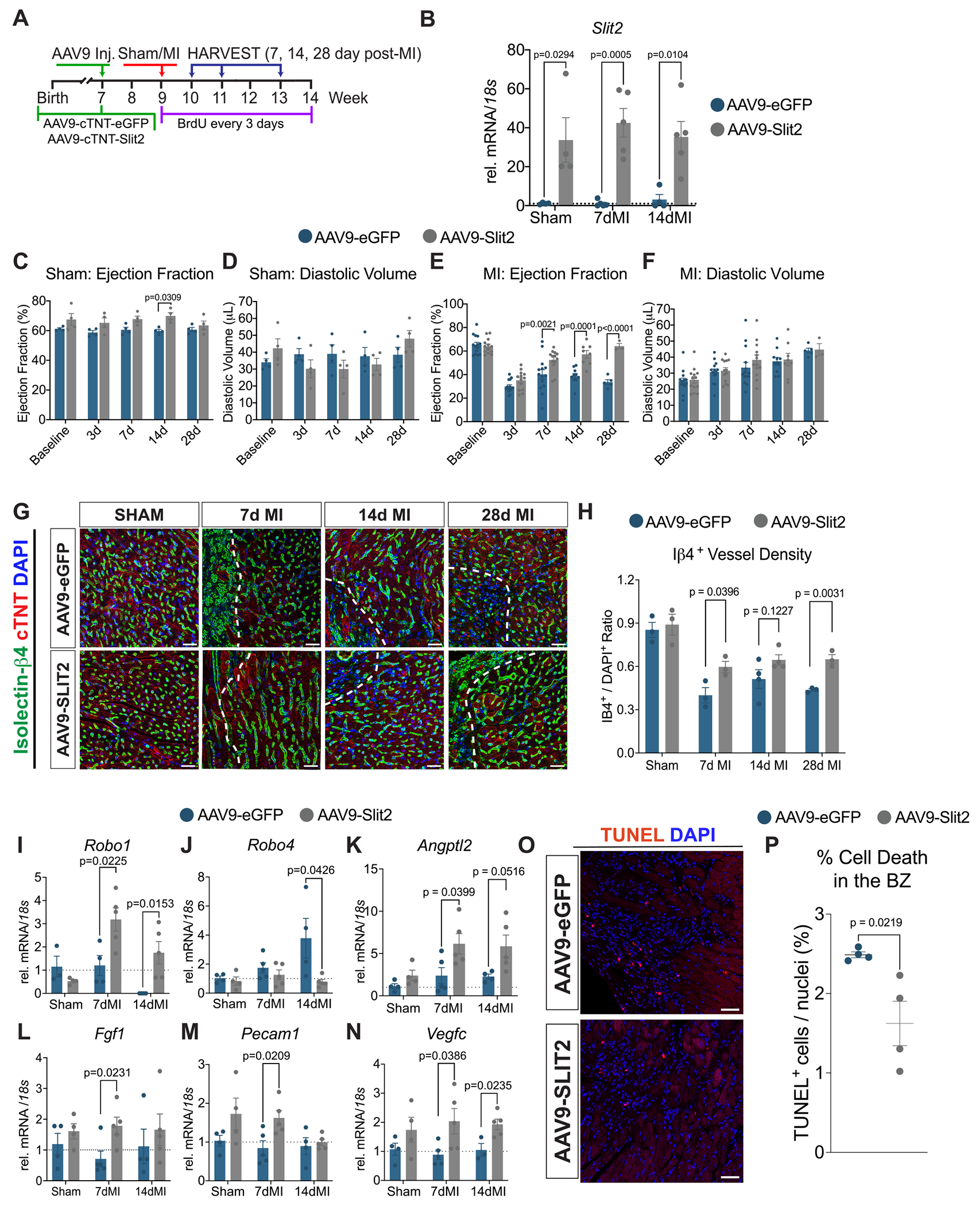
AAV9-mediated gene delivery of Slit2 improves cardiac function following myocardial infarction. A, Schematic of experimental to analyze mice following the injection of AAV9-cTNT-Slit2 and AAV9-cTNT-eGFP into *C57BL/6 J* mice 2 weeks before sham or myocardial infarction (MI) surgery. BrdU was injected every 3 days after injury, and echocardiography analyses were performed at baseline and 3-, 7-, 14-, and 28 days post-surgery. Tissue harvesting for gene expression of immunohistochemical analyses was performed at 7-, 14-, and 28 days post-surgery. B, *Slit2* in sham, 7- and 14-day post-MI hearts following AAV9-treatment. Sham/7dMI/14dMI AAV9-GFP (*N*= 4/*N*= 5/N = 4) and AAV9-Slit2 (N = 4/N = 5/N = 5) groups. An unpaired student’s *t*-test determined *P* values between each time point. C, Ejection fraction, and D, Diastolic volume of AAV9-treated mice subjected to sham surgery for up to 28 days. Baseline/3d/7d/14d/28d AAV9-GFP and AAV9-Slit2 N = 4 per group. P values were determined by Two-Way ANOVA with Sidak post-hoc test. E, Ejection fraction, and F, Diastolic volume of AAV9-treated mice subjected to MI surgery for up to 28 days. Baseline/3d/7d/14d/28d AAV9-GFP (*N* = 14/N = 14/N = 14/*N* = 10/N = 5) and AAV9-Slit2 (*N* = 13/N = 13/N = 13/*N* = 8/*N* = 3). Subsets of mice were sacrificed at 7- and 14-day post-MI for molecular and immunohistochemical analyses. P values were determined by Two-Way ANOVA with Sidak post-hoc test. G, Visualization of vessel density following immunofluorescence-based analysis of isolectin-B4 to label vascular cells in the left ventricle of sham hearts or border zone region following 7-, 14-, and 28-days post-MI and AAV9-treatment. Cardiac troponin T (cTNT, red) was utilized to label the myocardium, and a DAPI stain was used to label nuclei (blue). Scale bar, 20 μm. H, Quantification of vessel number based on Isolectin-β4 based fluorescence shown in (G). Representative images of histological sections from the indicated time points. Data indicates the average Isolectin-β4^+^ vessel count for at least 3 images for an individual mouse. Sham/7dMI/14dMI/28dMI AAV9-GFP (*N* = 4 for all time points) and AAV9-Slit2 (N = 4/N = 4/N = 5/N = 5). An unpaired student’s t-test determined P values between each time point. Expression of vascular/angiogenic genes I, *Robo1,* J, *Robo4,* K, *Angptl2*, L, *Fgf2*, M, *Pecam1,* N, *Vegfc* in tissue acquired from sham, 7-, and 14-day post-MI AAV9-treated mice. Sham/7dMI/14dMI AAV9-GFP (N = 4/N = 5/*N* = 4) and AAV9-Slit2 (N = 4/N = 5/N = 5) groups. An unpaired student’s *t*-test determined *P* values between each time point. O, Representative images of TUNEL (red) and DAPI (blue) staining in heart sections from AAV9-treated mice at 7 days post-MI. Scale bar, 20 μm. P, Quantification of TUNEL-positive nuclei as a percentage of total DAPI-stained nuclei in control (AAV9-eGFP) and AAV9-Slit2 injected hearts (*N* = 3 per group). Data were presented as mean +/− standard error of the mean (SEM).

Global SLIT2 overexpression confers cardioprotective effects during ischemia-reperfusion injury [19]. However, it remains unclear whether the observed improvements in myocardial function in SLIT2 transgenic mice were attributable to enhanced SLIT2 expression in the heart or if they resulted from off-target effects in other tissues. Given that Slit2 modulates angiogenesis during cardiac development, we investigated whether myocardial-targeted Slit2 could enhance vascular-driven cardiac repair following ischemic injury. To evaluate the functional effects of Slit2 in vivo, we generated an AAV9 vector encoding mouse SLIT2 under the control of the cTNT promoter (AAV9-cTNT-Slit2) and used an AAV9-cTNT vector expressing enhanced green fluorescent protein (AAV9-cTNT-eGFP) as a control (Fig. 4A). Although Slit2 is expressed in cFBs, viral vectors like AAV9 that use fibroblast-specific promoters have limited availability and require further validation. In contrast, the cTNT promoter limits transgene expression to the myocardium, enabling the assessment of cardiac-specific effects of secreted Slit2. AAV9-eGFP or AAV9-Slit2 viruses were injected retro-orbitally into 7-week-old *C57BL/6 J* mice, followed by either sham or MI surgery at 9 weeks of age (Fig. 4A). Bromodeoxyuridine (BrdU) was administered every 3 days to assess cell proliferation, and serial echocardiography was conducted at 3-, 7-, 14-, and 28 days post-injury. We performed an unbiased random selection of mice to complete tissue harvesting at 7, 14, and 28 days for gene expression and/or immunohistochemical analyses (Fig. 4A). SLIT2 overexpression was confirmed by RT-qPCR in sham and post-MI hearts (Fig. 4B). No significant differences in ejection fraction or diastolic volume were observed between AAV9-eGFP and AAV9-Slit2 sham-operated mice, although a modest, transient increase in ejection fraction was noted in AAV9-Slit2 mice 14 days post-Sham (Fig. 4C). Following MI, both AAV9-eGFP and AAV9-Slit2-treated mice showed a reduction in ejection fraction at 3 days post-MI; however, AAV9-Slit2-treated mice exhibited significantly improved cardiac function beginning at 7 days post-MI and persisting for up to 28 days (Fig. 4E). Despite increases in cardiac function in AAV9-Slit2 mice, no changes in diastolic volume (Fig. 4F) or collagen deposition in the left ventricle were observed (Supplemental Fig. 5A, 5B). Similarly, expression of ECM-related genes (*Col1a1*, *Col3a1*, *Fn*, *Postn*) did not differ significantly between control and AAV9-Slit2-treated groups in both sham and MI conditions (Supplemental Fig. 5C–F). Overall, our data suggest that increases in cardiac function following Slit2 overexpression are not mediated by changes in the ECM.

### 3.5. Overexpression of Slit2 induces angiogenic gene programs during ischemic remodeling

To evaluate the effects of cardiac-targeted Slit2 overexpression on angiogenic remodeling, we assessed vascular cell density and angiogenic gene expression in both sham and infarcted hearts. Isolectin-β4 staining, revealed a significant increase in vessel density in the BZ areas of 7- and 28-day post-MI AAV9-Slit2-treated mice compared to controls (Fig. 4G, H). Notably, *Robo1* expression was significantly upregulated in AAV9-Slit2-treated hearts at 7- and 14-day post-MI (Fig. 4I). *Robo4*, which was upregulated in AAV9-eGFP hearts at 14 days post-MI, was markedly suppressed by Slit2 overexpression (Fig. 4J). These data suggest that increases in myocardial Slit2 activate *Robo1* and suppress *Robo4*, the latter known to limit angiogenesis [11]. Consistent with this, expression of angiogenic genes, including *Angptl2*, *Fgf1*, *Pecam1,* and *Vegfc,* were elevated in AAV9-Slit2-treated hearts during early post-MI remodeling (Fig. 4K–N). Additionally *Angpt1*, *Aplnr*, *Pdgfa,* and *Tie2* were modestly upregulated in Slit2-overexpressing hearts compared to controls during acute MI (Supplemental Fig. 5G–J). Consistent with cardiac function improvements, Slit2-overexpressing hearts displayed significantly reduced terminal deoxynucleotidyl transferase dUTP nick end labeling (TUNEL) nuclei in the infarct and border zone regions, suggesting that enhanced angiogenesis in AAV9-Slit2 mice may contribute to improved cell survival (Fig. 4O–4P).

**Fig. 5.**
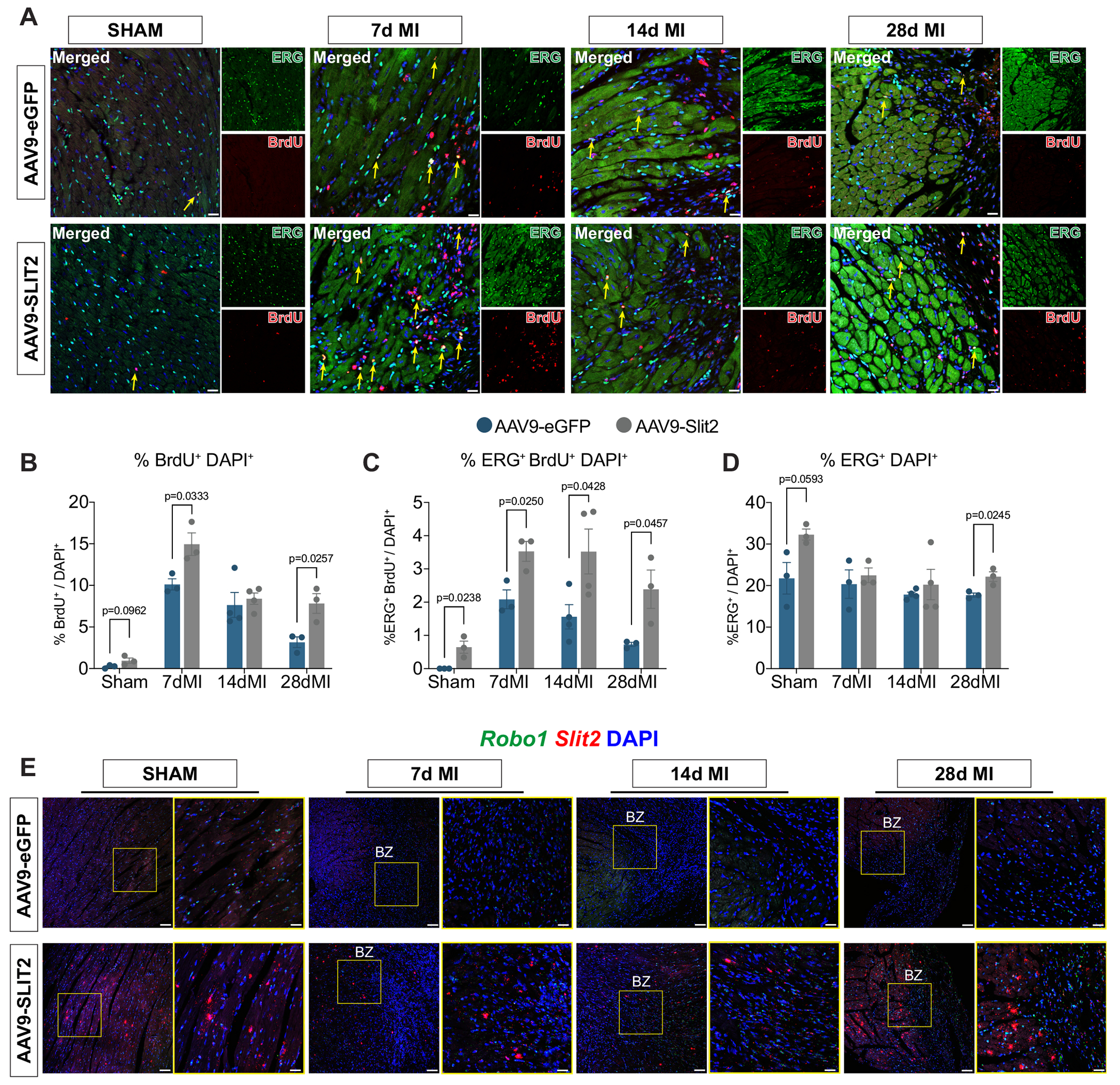
Slit2 overexpression promotes EC proliferation and Robo1 expression in the border zone after myocardial infarction. A, Heart sections from sham, 7-day, 14-day, and 28-day post-MI AAV9-treated mice. Representative images of sections labeled with antibodies against ERG (ETS-related gene, green) and BrdU (red). Nuclei were visualized with a DAPI stain (blue). Scale bar, 20 μm. B, Quantification of BrdU^+^, C, ERG^+^ and BrdU^+,^ and D, ERG^+^ cells normalized to the total number of DAPI^+^ nuclei represented in images shown in (A). Data indicates the average percentage (%) from at least 3 images for an individual mouse. Sham/7dMI/14dMI/28dMI AAV9-GFP and AAV9-Slit2 (*N* = 3/N = 3/*N* = 4/N = 3) groups. E, *Slit2* (red), and *Robo1* (green) expression determined by fluorescence in situ hybridization in AAV9-GFP and AAV9-Slit2 mouse hearts. DAPI staining was used to visualize nuclei (blue). White arrows, *Robo1*^+^ *Slit2*^+^ cells. *N* = 3 biological samples imaged at each age. Scale bar, 20 μm. Data were presented as mean +/− standard error of the mean (SEM). (For interpretation of the references to colour in this figure legend, the reader is referred to the web version of this article.)

To determine whether increases in angiogenic gene programs contribute to changes in EC proliferation, we measured BrdU incorporation in ETS transcription factor (ERG)-positive cells (Fig. 5A). As expected, BrdU^+^ cell frequency increased in both control and AAV9-Slit2-treated MI hearts compared to sham (Fig. 5B). However, AAV9-Slit2-treated hearts displayed significantly more BrdU^+^ cells at both 7- and 28-day post-MI (Fig. 5B), along with increased BrdU incorporation specifically in ERG^+^ ECs at all MI time points compared to controls (Fig. 5C). This increase in proliferating ECs correlated with a significant rise in overall ERG^+^ cell density in AAV9-Slit2 hearts at 28 days post-MI (Fig. 5D). Additionally, Slit2 overexpression in mice subjected to MI displayed increased co-localization of *Slit2* and *Robo1*, suggesting enhanced receptor-ligand interactions (Fig. 5E). We next assessed immune cell infiltration and pro-inflammatory gene expression 7 days post-MI to determine whether the improved EC proliferation was accompanied by alterations in the inflammatory response. Slit2 overexpression resulted in a modest reduction in CD68^+^ macrophage infiltration and no differences in pro-inflammatory cytokine expression, suggesting that its pro-angiogenic effects occur independently of heightened inflammatory activation (Supplemental Figs. 5K,5L, and 6A, 6B). Additionally, fibrinogen deposition near Isolectin-β4^+^ vasculature - a marker of vascular leakage and plasma protein extravasation - was not significantly altered in mice 7 days post-MI (Supplemental Fig. 6C, 6D), suggesting that vessels in AAV9-Slit2 mice are stable.

Collectively, these results indicate that myocardial Slit2 overexpression during ischemic remodeling enhances angiogenic gene expression and promotes EC proliferation, contributing to improved vascularization and cardiac repair.

### 3.6. Slit2 attenuates cardiomyocyte hypertrophy in vivo and in vitro

**Fig. 6.**
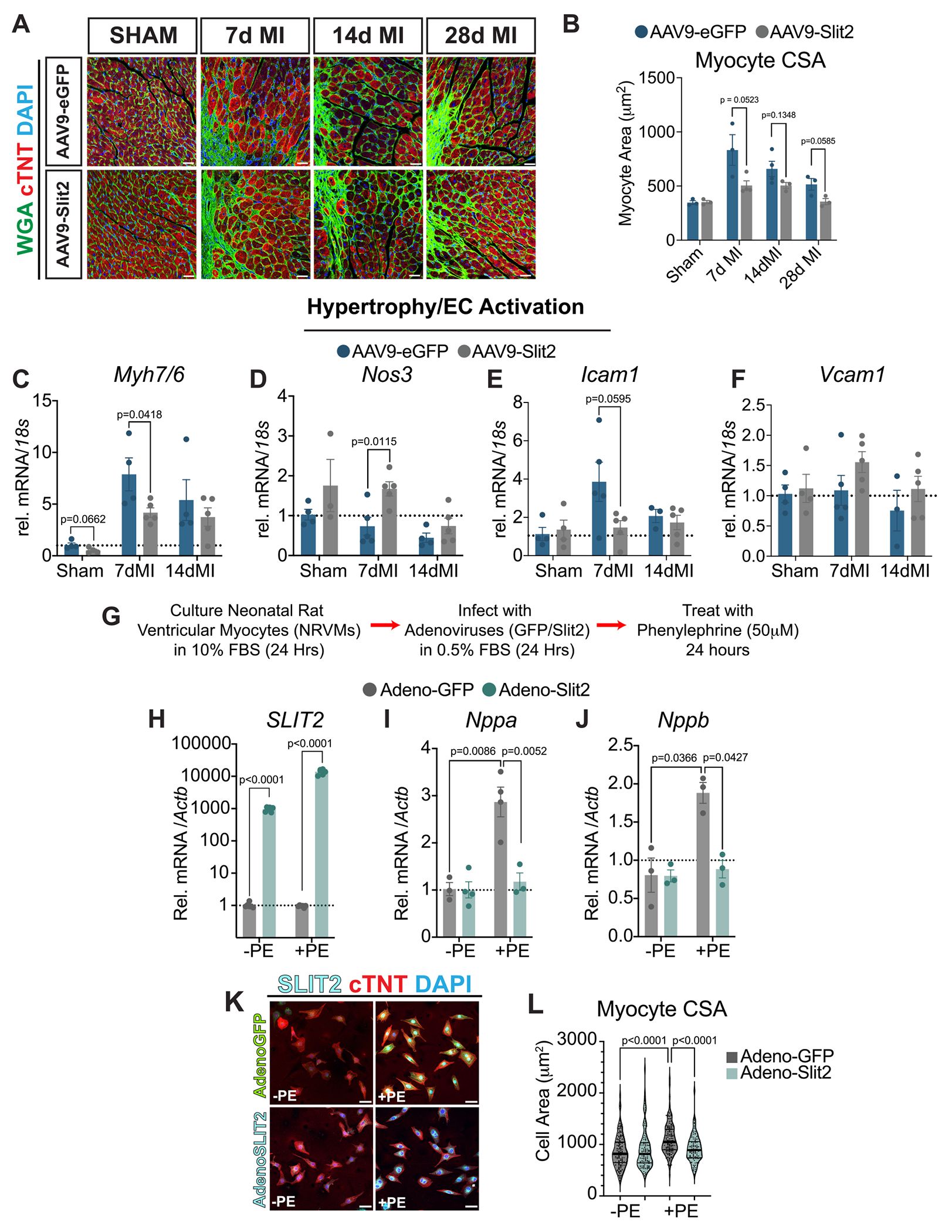
Overexpression of Slit2 in the myocardium inhibits cardiomyocyte hypertrophy. A, Visualization of the cross-sectional area (CSA) of cardiomyocytes following immunofluorescence-based analysis of wheat germ agglutinin (WGA) labeling of cellular membranes in the left ventricle of sham hearts or border zone region following 7-, 14-, and 28-days post-MI and AAV9-treatment. Cardiac troponin T (cTNT, red) was utilized to label the myocardium, and a DAPI stain was used to label nuclei (blue). Scale bar, 20 μm. B, Quantification of cardiomyocyte CSA represented in (A). Data indicates the average cardiomyocyte CSA for at least 3 images for an individual mouse. Sham/7dMI/14dMI/28dMI AAV9-GFP (N = 3/N = 3/N = 4/N = 3) and AAV9-Slit2 (N = 3/N = 4/N = 3/N = 3). An unpaired student’s *t*-test determined *P* values between each time point. Expression of cardiomyocyte hypertrophy genes C, *Mhy7/Mhy6* ratio*,* and endothelial activation genes D, *Nos3,* E, *Icam1*, and F, *Vcam1* in tissue acquired from sham, 7-, and 14-day post-MI AAV9-treated mice. Sham/7dMI/14dMI AAV9-GFP (N = 4/*N* = 5/N = 4) and AAV9-Slit2 (N = 4/N = 5/N = 5) groups. An unpaired student’s t-test determined P values between each time point. G, Schematic of experimental design to culture neonatal rat ventricular cardiomyocytes (NRVMs), infect with adenoviruses for 24 h, and treat with pro-hypertrophic stimulus phenylephrine (PE) for an additional 24 h. H, *SLIT2*, I, *Nppa*, and J, *Nppb* in NRVMs infected with Adeno-GFP or Adeno-Slit2 determined by RT-qPCR. *N* = 3 per group and condition. An unpaired student’s *t*-test determined *P* values between each time point/condition. K, Immunocytochemical analysis of NRVMs with or without PE, visualized by labeling with cTNT and DAPI. Exogenous GFP and SLIT2 expression following adenoviral-based infection are represented in green and teal, respectively. Scale bar, 50 μm. L, Quantification of NRVM CSA from images shown in (K). Data indicates the average NRVM CSA for at least 3 images for an individual mouse. N = 3 per group/condition. An unpaired student’s t-test determined P values between groups/conditions. Data were presented as mean +/− standard error of the mean (SEM). (For interpretation of the references to colour in this figure legend, the reader is referred to the web version of this article.)

Slit2 overexpression increases contractility in CMs following ischemia-reperfusion injury, although its effects on CM hypertrophy were not assessed [19]. Both AAV9-eGFP and AAV9-Slit2-treated mice exhibited increased hypertrophic remodeling after MI, as measured by wheat germ agglutinin (WGA) staining to quantify cardiomyocyte crosssectional area (CSA) (Fig. 6A, B). However, AAV9-Slit2-treated mice displayed a significant reduction in CM hypertrophy at 7- and 28-days post-MI in the border zone regions (Fig. 6B), with no differences observed in the remote myocardium (Supplemental Fig. 5M, 5N). This attenuation of hypertrophy was accompanied by a decrease in the fetal gene ratio *Mhy7*/*Mhy6* compared to controls (Fig. 6C). Additionally, *Nos3* expression was markedly upregulated in AAV9-Slit2-treated mice 7 days post-MI (Fig. 6D), coinciding with decreased *Icam1* expression, while *Vcam1* levels remained unchanged (Fig. 6E, F). These findings are consistent with prior studies linking increased Nos3 to improved ventricular function [35] and reports that Slit2 inhibits ICAM-1 in ECs during inflammatory-induced stress [36].

To further evaluate SLIT2’s effects on hypertrophic signaling, we treated neonatal rat ventricular myocytes (NRVMs) with adenoviruses encoding either SLIT2 or GFP (control), followed by treatment with the α-adrenergic agonist phenylephrine (PE) to induce hypertrophy (Fig. 6G). SLIT2 overexpression in NRVMs was confirmed in vehicle and PE-treated cells relative to Adeno-GFP controls (Fig. 6H). Treatment of Adeno-GFP myocytes with PE led to a robust induction of fetal hypertrophic genes, *Nppa* and *Nppb*, which were both significantly attenuated in SLIT2-overexpressing NRVMs (Fig. 6I, J). Immunocytochemistry further confirmed reduced CM size in Adeno-Slit2 and PE-treated NRVMs compared to controls (Fig. 6K, L). Although previous studies have shown that *Robo1* and *Robo4* are primarily expressed in fibroblasts and ECs, respectively, recent work suggests that SLIT3 can regulate CM hypertrophy via ROBO1 signaling [37]. To examine the distribution of Slit2-Robo components, we analyzed single-nucleus RNA-sequencing data from young and aged murine hearts [38]. *Slit2* and *Slit3* were enriched in cFBs (Supplemental Fig. 7A, 7B), while *Robo1* was primarily expressed in cFBs, with lower levels in CMs. In contrast, *Robo2* and *Robo4* expression was restricted to ECs (Supplemental Figure 7A, 7C). These data indicate that Slit2 mediates its effects on hypertrophic remodeling primarily through Robo1 signaling in fibroblasts, and potentially through ECs. Although we show conclusively that Slit2 attenuates cardiomyocyte growth, this effect does not appear to be exclusively dependent on Robo1, suggesting that alternative or compensatory pathways may also contribute.

### 3.7. Slit2 regulates the secretome of cardiac fibroblasts and enhances the activation and proliferation of endothelial cells

**Fig. 7.**
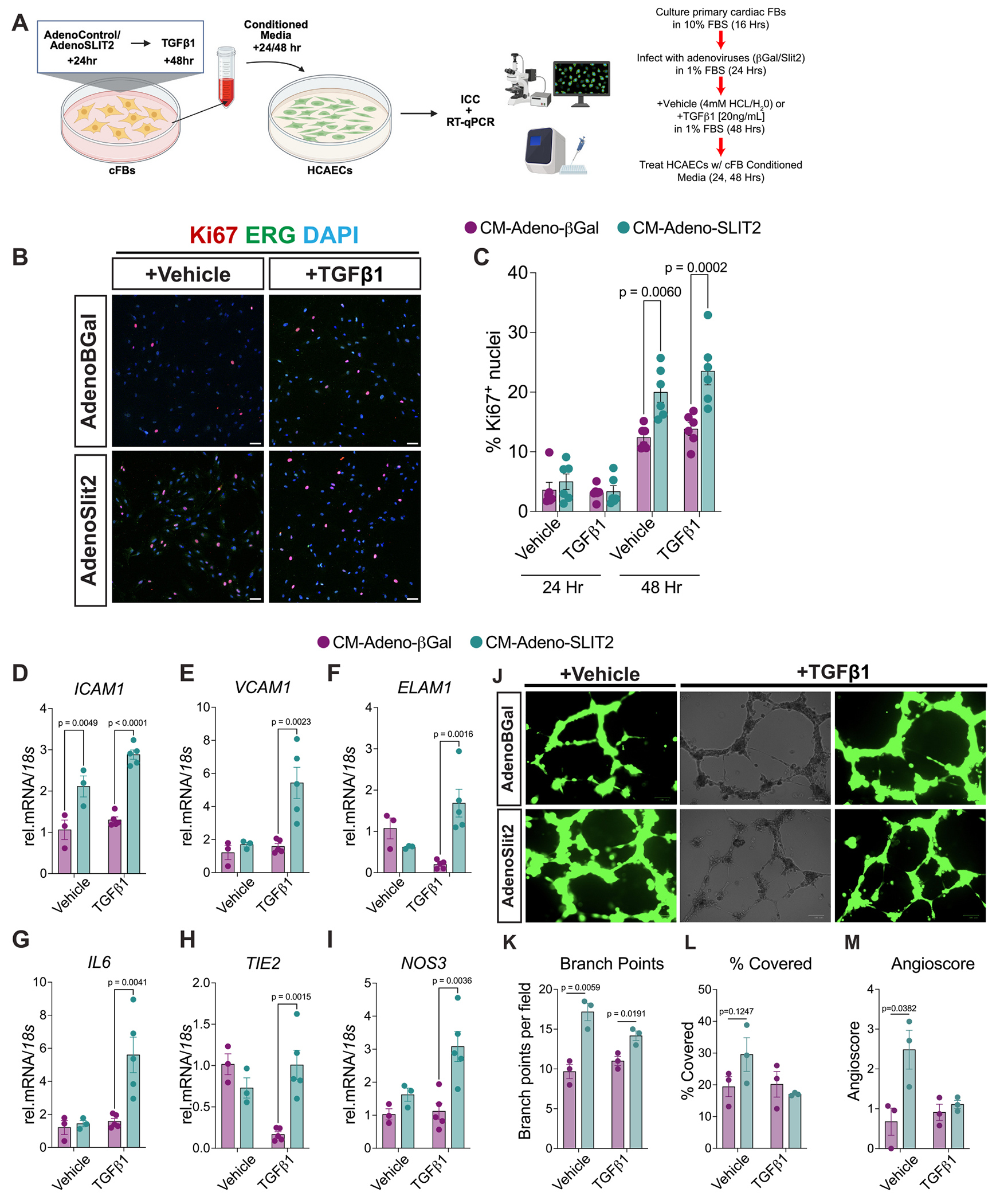
Slit2 regulates the secretome of cardiac fibroblasts and enhances the activation and proliferation of endothelial cells. A, Schematic overview of experimental workflow. Primary cardiac fibroblasts were cultured and infected with adenovirus expressing Slit2 (AdenoSlit2) or β-Galactosidase (AdenoβGal), followed by treatment with vehicle or TGFβ1 for 48 h. Conditioned cFBs media were used to treat human coronary artery endothelial cells (HCAECs) for 24 or 48 h. B, Representative immunofluorescence images of HCAECs treated with conditioned media from cFBs overexpressing Slit2 or βGal, in the presence or absence of TGFβ1. Antibodies labeled for Ki67 (red), ERG (green), and DAPI (nuclei, blue). Scale bar: 50 μm. C, Quantification of Ki67^+^ nuclei in HCAECs after treatment with conditioned media for 24 or 48 h. (*N* = 6 per condition). D–I, Gene expression analysis of endothelial cell activation markers *ICAM1*, *VCAM1, ELAM1, IL6, TIE2,* and *NOS3* in HCAECs treated with conditioned media for 48 h. N = 3 for vehicle-treated, N = 5 for TGFβ1-treated. J, Representative fluorescent and brightfield images of endothelial cell tube formation in Matrigel following infection with AdenoβGal or AdenoSlit2 and treatment with or without TGFβ1 for 12 h. Scale bar = 100 μm. K-M, Quantification of HCAEC angiogenic metrics, including total number of branch points (K), percentage of Matrigel field of view covered (L), and Angioscore ((*Z*-score of branch points + Z-score of % area covered) / 2) – minimum value (across all samples) (M). each dot represents an independent well, with 3 fields taken per well. An unpaired student’s t-test determined P values between conditions. Data were presented as mean +/− standard error of the mean (SEM). (For interpretation of the references to colour in this figure legend, the reader is referred to the web version of this article.)

We next sought to determine if SLIT2-overexpressing cFBs, with or without TGFβ1-induced myofibroblast differentiation, could modulate cross-talk with ECs. To test this, we collected conditioned media from primary mouse cFBs infected with Adeno-βGal or Adeno-Slit2 for 24 h before treatment with vehicle or TGFβ1 for 48 h (Fig. 7A). Conditioned media from all four groups were applied to human coronary artery endothelial cells (HCAECs) for 24 or 48 h (Fig. 7A). Conditioned media from Adeno-Slit2-treated cFBs significantly increased EC proliferation, as indicated by Ki67^+^ nuclei, compared to controls after 48 h of treatment (Fig. 7B, C). Additionally, conditioned media from Slit2-expressing cFBs increased the expression of EC activation genes *ICAM1, VCAM1, ELAM1*, *IL6*, *TIE2, and NOS3* (Fig. 7D–I), suggesting that Slit2 enhances EC activation through fibroblast-derived paracrine signals. Functional angiogenesis assays further revealed that conditioned media from Slit2-overexpressing cFBs significantly enhanced EC sprouting, as evidenced by increased branch points and angiogenic scores compared to control-treated HCAECs (Fig. 7J–M). The pro-angiogenic abilities of ECs were reduced when treated with conditioned media from cFBs stimulated with TGFβ1, indicating that myofibroblast differentiation modifies the angiogenic potential of the Slit2-conditioned secretome in vitro.

To further characterize how SLIT2 alters the cFB secretome, we screened the conditioned media from Slit2-overexpressing cFBs using a protein array to detect 111 cytokines. Fibroblasts treated with TGFβ1 showed increased CCL17, IL-33, CCN4, and CX3CL1 in their conditioned media in Adeno-Slit2 and control groups (Supplemental Fig. 8A). Conditioned media from the Adeno-Slit2 cFB vehicle treatment group showed upregulated IGFBP6 and CCL17, as well as Osteopontin and IL-33 following TGFβ1, compared to controls under the same treatment (Supplemental Fig. 8B, 8C). Osteopontin has been shown to regulate angiogenesis by activating VEGF and PI3K/AKT signaling [39]. To determine whether conditioned media from Slit2-overexpressing cFBs increased the phosphorylation of angiogenic factors, we visualized the phosphorylation status of VEGFR2 (Tyr1175) and AKT (Ser473) using immunocytochemistry. We found no significant changes in phosphorylation after 48 h of treating HCAECs (Supplemental Figs. 8D and 8E), suggesting that signaling may occur more acutely in vitro. Collectively, these results indicate that SLIT2 alters the fibroblast secretome to enhance EC proliferation and activation, highlighting a novel role for SLIT2 in the interaction between fibroblasts and ECs.

## 4. Discussion

### 4.1. Slit-robo signaling across development, aging, and disease

Slit2 is a key vascular growth and maturation regulator through its differential interactions with Robo1 and Robo4 [1,11,40,41]. Previous studies from our lab have shown that Slit2 is highly enriched in epicardial-derived fibroblasts during coronary plexus remodeling, co-localizing with Robo4^+^ ECs [1]. Additionally, adenoviral-mediated overexpression of Slit2 in the distal epicardium altered the expression of venous and angiogenic gene markers in ECs, indicating that Slit2 is sufficient to influence EC fate specification during cardiac development [1]. These findings emphasize an essential Slit2-mediated paracrine signaling mechanism between the epicardium and developing coronary vasculature that alters cardiac morphogenesis.

In the present study, we extend these findings during development by examining the endogenous expression and function of Slit2 in the adult heart and during cardiac repair resulting from myocardial ischemia. Through transcriptional sequencing and biological validation, we found that Slit2 is highly enriched in cFBs during early postnatal stages but declines significantly with age. This age-associated reduction mirrors previous studies of adult human epicardial cells, which display diminished paracrine signaling and reduced regenerative and angiogenic gene programs compared to fetal epicardial cells [7]. Interestingly, although Slit2 expression was transiently increased during the first week post-MI, it rapidly declined during chronic ischemia, suggesting an insufficient endogenous response to injury. This pattern of reduced Slit2 expression in the adult and chronic ischemia supports our hypothesis that the limited upregulation of pro-angiogenic molecules, such as Slit2, hinders effective cardiac repair.

**Fig. 8.**
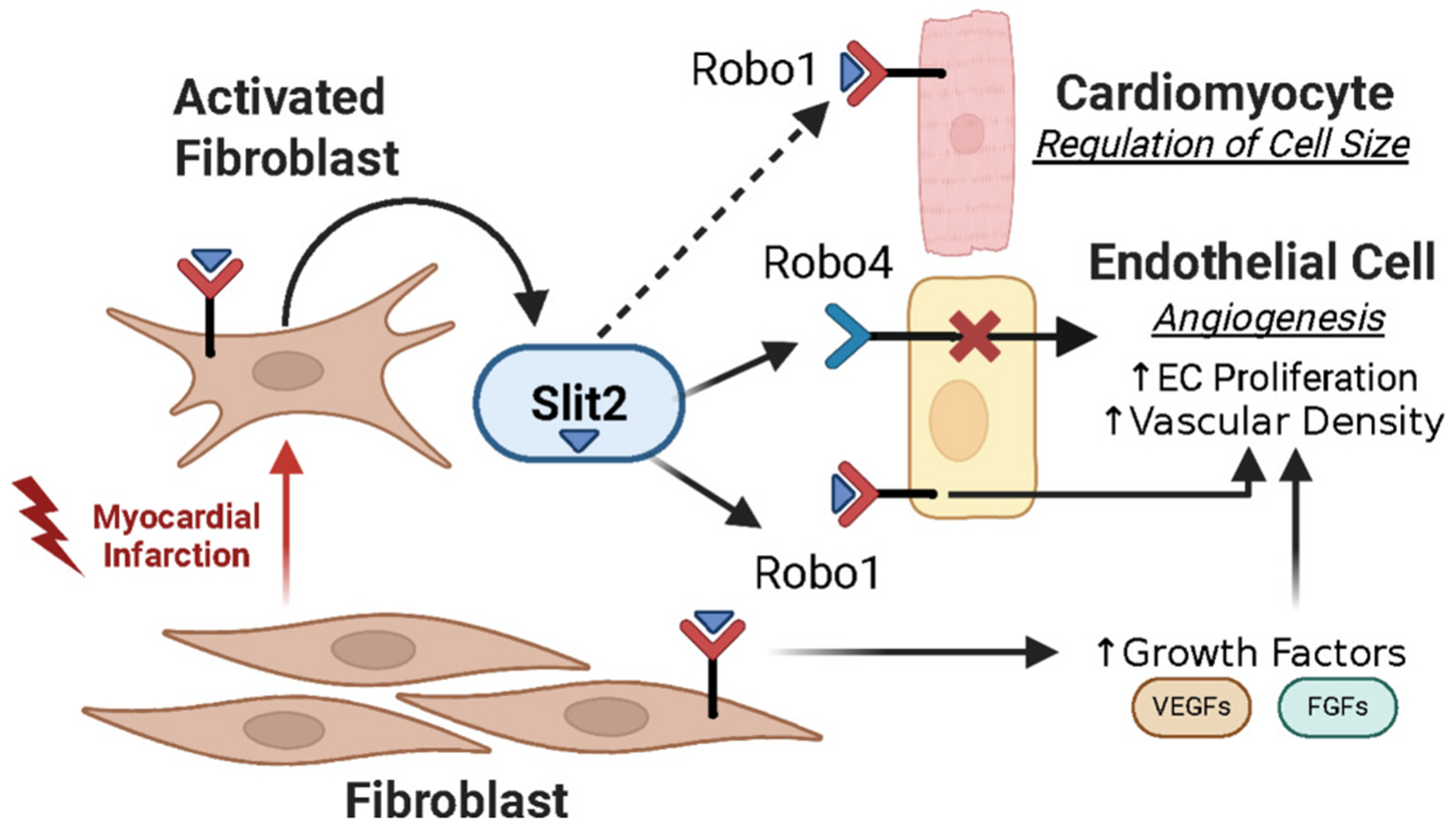
Proposed Mechanism of Slit2-Mediated Cardiac Repair Post-Myocardial Infarction. Myocardial infarction (MI) triggers the activation of fibroblasts, which in turn upregulate Slit2 expression. Secreted Slit2 from cardiac fibroblasts interacts with Robo1 receptors on fibroblasts and endothelial cells to promote angiogenic signaling pathways. Slit2 also attenuates cardiomyocyte hypertrophy in MI- and phenylephrine-induced models; however, it is unclear whether this effect is mediated through Robo1. The dashed line indicates a potential correlative relationship between Slit2 and Robo1 in cardiomyocytes.

During acute MI, we observed elevated levels of *Robo1* and *Robo4* in the adult heart. Analysis of transcriptional sequencing datasets of healthy and MI hearts revealed that *Robo1* was significantly enriched in cFBs, with lower levels detected in CMs and ECs. In contrast, *Robo4* was expressed exclusively by ECs. Although Robo1 and Robo2 transcripts were detected in rare EC subsets, their limited expression contrasts sharply with the widespread and robust expression of Robo1 on fibroblasts. Previous studies suggest that Robo1 and Robo4 may exert distinct, and in some cases antagonistic, roles in vascular biology, underscoring the importance of cell- and context-specific Slit2-Robo interactions in post-ischemic remodeling. While it remains unclear whether Slit2 mediates its cardioprotective effects through interactions with Robo1, we note that Slit2 overexpression during ischemiareperfusion results in elevated Robo receptor expression similar to that observed in our MI model – namely, upregulation of Robo1 and suppression of Robo4 (Fig. 8). Given the distinct expression patterns of Robo1 and Robo4 across cardiac cell types, further exploration of cell-specific Slit-Robo signaling could uncover new strategies for improving cardiac repair.

### 4.2. Differential roles of cardiac fibroblasts in post-ischemic remodeling

Recent high-throughput transcriptomic studies have highlighted the heterogeneity of cFB populations, with distinct subsets serving multiple functions during development, disease progression, and tissue repair [42–45]. However, the specific fibroblast subtypes that regulate non-canonical processes, such as angiogenesis, remain poorly understood [46]. In this study, adenovirus-mediated Slit2 overexpression in cFBs selectively induced the expression of pro-angiogenic ligands, including VEGF and FGFs, without altering the expression of ECM-related genes. Few studies have explored the pro-angiogenic properties of cFBs in vivo after cardiac injury. One study reported that fibroblasts secrete pro-angiogenic factors, such as SLIT2, EPHB4, and VEGFA, at 3 days postMI, but by 7 days post-MI, anti-angiogenic factors become upregulated, highlighting the transient and dynamic nature of the angiogenic response of fibroblasts in the heart [5,47]. These findings underscore the dual role of fibroblasts in coordinating both fibrotic and angiogenic programs after MI, adding to the complexity of post-injury cardiac remodeling. Future studies using fibroblast-specific Cre drivers (*Tcf21-Cre or Postn-Cre)* may help elucidate if the fibrotic/angiogenic response of cFBs is dependent on Slit2 signaling and uncover new strategies to enhance vascular repair while limiting fibrosis following MI.

### 4.3. AAV9-Slit2 enhances post-MI recovery

Our study demonstrates that AAV9-mediated overexpression of Slit2 significantly improves cardiac function after MI, likely through the enhancement of angiogenic signaling. Previous work has indicated that SLIT2 exerts cardioprotective effects in ischemia-reperfusion injury [19]; however, the impact on vasculogenesis was not assessed. By employing a cardiac-specific AAV9-cTNT-Slit2 vector, we isolated the effects of Slit2 within the heart and demonstrated its role in activating vascular gene programs. In contrast to Robo1, which was upregulated by Slit2 overexpression, Robo4 was suppressed in hearts treated with AAV9-Slit2. This differential Robo receptor expression pattern in response to AAV9-mediated Slit2 overexpression is similar to that of transgenic mice overexpressing Slit2 globally during ischemiareperfusion injury [19]. Our data indicate that Slit2-Robo1 interactions in cFBs promote angiogenic gene expression, while Slit2-Robo4 in endothelial cells may promote vascular stability through the inhibition of ARF6, a GTPase that activates Rac/Rho signaling [11]. Interestingly, we observed differential effects of Slit2 overexpression on ICAM1 in vitro and in vivo. While conditioned media from Slit2-overexpressing fibroblasts increased *ICAM1* in cultured ECs, cardiacspecific overexpression of Slit2 in vivo reduced *ICAM1* in the heart post-MI. This discrepancy likely reflects context-dependent differences and the reliance on Slit2-Robo4 signaling. In vitro, fibroblast-derived paracrine factors may promote EC activation without cellular and tissue microenvironment regulatory cues. In vivo, Slit2 binding to Robo4 on ECs upregulates ICAM1 and other inflammatory cytokines/chemokines [36]. We observed that Robo4 was reduced in AAV9-Slit2-treated hearts, consistent with a reduction in *Icam1*, suggesting a Slit2-Robo4-dependent mechanism that modulates EC activation and function during cardiac remodeling. Altogether, these findings suggest a coordinated role for Slit2 signaling through Robo1 and Robo4, and future studies examining the effects of Slit2 overexpression following knockout of cell-specific Robo1/4 may help elucidate the precise mechanism by which Slit2 improves cardiac repair.

In addition to enhancing vascular cell density, sustained Slit2 expression attenuated CM hypertrophy, indicating that the transient increase in endogenous Slit2 during the acute stages of MI may help to constrain compensatory pathological hypertrophic growth [48]. Notably, the effects of Slit2 on CM growth contrast with *Slit3*, which also binds and uses Robo1 as a receptor, but promotes hypertrophy during pressure overload stress [37]. Although the exact signaling mechanisms downstream of Slit2- or Slit3-Robo1 interactions remain undefined in the heart, our transcriptional data suggest that Slit2 likely does not act directly on CMs, which express low levels of Robo1 as compared to fibroblasts [18]. Instead, we hypothesize that increases in vascular growth and related gene programs, likely accompanied by enhanced perfusion, contribute to improvements in CM function. However, we cannot rule out the possibility of reciprocal signaling between myocardial and vascular compartments that may further reinforce tissue repair during ischemia (Fig. 8).

This is the first study to define a functional role of Slit2 in the adult heart, particularly after ischemic injury. Increasing Slit2 levels beyond its limited endogenous induction post-MI confers therapeutic benefit, primarily through vascular and fibroblast-mediated mechanisms involving Robo1. As fibroblast electrical coupling and paracrine crosstalk with CMs are well-documented in both homeostasis and disease, particularly following MI [49,50], it remains possible that fibroblasts sourced Slit2 modulates broader cellular networks critical for maintaining myocardial integrity. Ultimately, deepening our understanding of Slit2-mediated signaling downstream and across cell types may offer novel strategies to enhance cardiac repair and improve outcomes in ischemia-related cardiac disease.

## Supplementary Material

Supplemental Material

## Appendix A. Supplementary data

Supplementary data to this article can be found online at https://doi.org/10.1016/j.yjmcc.2025.10.014.

## Abbreviations:

AAV-9: Adeno-associated Virus Serotype 9
cFB: Cardiac Fibroblast
CM: Cardiomyocyte
cTNT: Cardiac Troponin T
EC: Endothelial Cell
ECM: Extracellular Matrix
HCAEC: Human Coronary Endothelial Cell
MI: Myocardial Infarction
Robo: Roundabout receptor
Slit: Slit guidance ligand
