# Supplementary material for: Slit2-robo signaling regulates angiogenesis and repair following myocardial infarction
Source: J Mol Cell Cardiol. Author manuscript; Available in PMC 2026 May 28. (PMC13218272; doi:10.1016/j.yjmcc.2025.10.014)
Supplement: Supplemental Material [file NIHMS2174291-supplement-Supplemental_Material.pdf]

Supplemental Material

Slit2-Robo Signaling Regulates Angiogenesis and Repair Following Myocardial Infarction

David Wong B.S.[1,2], Matthew Tran B.S.[1], Julie Martinez M.S.[1,2], Itzetl Avila B.S.[2,4], Adrian Arrieta Ph.D.[5], Kyle Kalindijan[6], Elle Rathbun B.S.[7], Thomas M. Vondriska Ph.D.[5], Eric M. Small Ph.D. [8], Pearl Quijada Ph.D.[1,9,10]*

Affiliations: [1]Department of Integrative Biology and Physiology, University of California, Los Angeles, [2]Molecular, Cellular, and Integrative Physiology Graduate Program, University of California, Los Angeles, [3]Department of Biology, University of California, Los Angeles, [4]Department of Microbiology, Immunology & Molecular Genetics, University of California, Los Angeles, [5]Department of Anesthesiology & Perioperative Medicine, David Geffen School of Medicine at the University of California, Los Angeles, [6]Computational & Systems Biology, University of California, Los Angeles, [7]Department of Neurology, David Geffen School of Medicine at the University of California, Los Angeles, [8]Aab Cardiovascular Research Institute, Department of Medicine, University of Rochester School of Medicine and Dentistry, [9]Eli and Edythe Broad Stem Cell Research Center, University of California, Los Angeles, and [10]Molecular Biology Institute, University of California, Los Angeles.

*Correspondence to: Pearl Quijada, Ph.D., Integrative Biology and Physiology, Division of Life Sciences, University of California, Los Angeles, 610 Charles E Young Drive S, Los Angeles, CA 90095, Email: pquijada@ucla.edu

The Supplemental Material file includes the following:
Supplemental Methods
Supplemental Tables S1-3
Supplemental Figures S1-8
Supplemental Material (Uncropped Immunoblot)
References 1-4

Supplemental Methods

Experimental animals

All animal studies, including husbandry, breeding, and experimental procedures, followed protocols approved by the University Committee on Animal Resources at the University of California, Los Angeles. The C57BL/6J mice strain (stock #000664) was obtained from the Jackson Laboratory. A C57BL/6J colony was maintained in our vivarium by crossing C57BL/6J male mice to C57BL/6J female mice. Female mice of the C57BL/6J stock mouse strain from the Jackson Laboratory were introduced into our in-house colony every 10 generations to prevent genetic drift. Wt1-CreERT2 heterozygous males were purchased from the Jackson Laboratory (stock #010912) and crossed to mT/mG homozygous females (stock #007676) to generate heterozygous Wt1-CreERT2; mT/mG embryos. Mice expressing the H2B-eGFP fusion gene from the endogenous *Pdgfra* locus (*PDGFRa*^{nGFP} mice) were purchased from the Jackson Laboratory (stock #007669). All mice were maintained in a C57BL/6J background and a temperature-controlled environment with 12-hour light/dark cycles, receiving food and water *ad libitum*.

Epicardial cell and heart isolations during embryonic development

Timed pregnancies were determined after placing one Wt1-CreERT2 heterozygous male with two mT/mG homozygous Cre reporter females in a single cage in the late afternoon. Additionally, to generate C57BL/6J embryos, one C57BL/6J male was placed with two C57BL/6J females. The confirmed plugs were timed as embryonic day (E) 0.5 the following morning, and the females were immediately separated from the males. To induce Cre-based recombination, 4-Hydroxytamoxifen (4-OHT, Millipore Sigma H6278) was dissolved in sunflower seed oil from helianthus annus (Millipore Sigma S5007) at a final concentration of 10 mg/mL with 10% ethanol. 4-OHT was administered by oral gavage at 75 mg/kg to pregnant dams. 4-OHT was administered at E9.5 and E10.5, and embryos (male and female) were isolated at E12.5, E14.5 and E16.5. On the day of isolation, pregnant dams

were anesthetized with an intraperitoneal injection of 0.5 mL of ketamine-xylazine cocktail (13 mg/mL ketamine in 0.88 mg/mL xylazine in DPBS), followed by cervical dislocation. After the use of 70% ethanol to sterilize the abdominal area, an incision to enter and remove decidua away from the mesometrium was performed, and embryos were placed in pre-warmed HBSS (ThermoFisher Scientific, SH30031.02). After removing extraembryonic tissue and the yolk sac, the heart was removed from the embryo and placed in a cell culture well containing HBSS. Digestion of embryonic hearts at E12.5, E14.5, and E16.5 began by removing residual HBSS from wells and replacing media with a digestion solution containing 0.08% Collagenase IV (Worthington Biochemical, LS004188), 0.05% Trypsin Protease (ThermoFisher Scientific, SH3004201), 1% fetal bovine serum (FBS) (ThermoFisher Scientific, SH3091003HI) diluted in pre-warmed HBSS before placing hearts in a 37°C hybridization oven with gentle shaking for 5 min intervals. Following incubation, hearts were dissociated by gentle pipetting (3-5 times with a P1000 pipette), and undigested tissue was allowed to settle for 30s. After tissue settlement, media was collected and added to a separate tube containing FBS to neutralize digestion, and digested cells were then saved on ice. Digestion, pipetting, and media collection were repeated 3-5 more times, and cells were then filtered through a 70μm filter and centrifuged at 200 × *g* for 5 min at 4 °C. The resulting pellet was placed in 0.5% bovine serum albumin (BSA) (Sigma-Aldrich, A9647) solution dissolved in sterile PBS and saved on ice before performing fluorescence-activated cell sorting (FACS) using a BD FACS Aria II and 100μm nozzle (BD Biosciences). DRAQ5, a far-red cell-permeant stain (Thermo Fisher Scientific [5mM], 62251), was added to cells immediately before sorting at a dilution of 1:10,000 in 0.5% BSA/PBS solution to exclude dead cells. Green fluorescent protein (GFP)-positive cells (*Wt1*-lineage) were sorted directly into 1.5 mL Eppendorf tubes containing 0.5% BSA/PBS solution at 4°C and immediately processed for downstream RNA isolation and gene expression assays using TRIzol-based cell lysis (Fisher Scientific, 15-596-018).

The protocol above was followed with modifications for isolating whole hearts at E16.5 from mice of the C57BL/6J mouse strain background, which did not require administration of 4-OHT. After removing HBSS from wells following the isolation of hearts into multi-well plates, the cells were immediately covered with 10% neutral-buffered formalin (Fisher Scientific, 22-050-105) and placed on a shaker for 24 hours at room temperature. After 24 hours, the hearts were placed in 70% Histology Grade Ethanol (Fisher Scientific, 22-050-109) and submitted for paraffin embedding and sectioning at the University of California, Los Angeles David Geffen School of Medicine Translational Pathology Core Laboratory (TPCL).

Neonatal mouse ventricular cardiomyocyte and cardiac fibroblast isolation

Hearts were isolated from neonatal (P1) C57BL/6J and *PDGFRa^{nGFP}* mouse pups (male and female) following hypothermia and decapitation. The atria and atrioventricular canal were removed, and the hearts were minced with scissors. The hearts were placed in digestion buffer [0.8 mg/mL Collagenase Type 2 (Worthington Biochemical, LS004174) dissolved in PBS and transferred to a scintillation vial containing a magnetic stirrer. The hearts were incubated at 37℃ on a magnetic stir plate for 50 minutes. The cells in the digestion solution were removed and replaced with fresh digestion buffer every 10 minutes. This was repeated 5 times with the digestion solution from the second to fifth rounds kept in normal growth cardiac fibroblast media containing DMEM (Corning, 10-17-CV) with 10% FBS, 1% penicillin- streptomycin (Fisher Scientific, SV30010), and 1% L-glutamine (Fisher Scientific, MT25005CI) for cell culture. The digestion solutions were pooled and filtered through a 70μm strainer, centrifuged, and the cell pellet was resuspended in normal growth cardiac fibroblast media. The cells were seeded on standard polystyrene cell culture plates for 2 hours, during which cardiac fibroblasts would attach to the plate. The supernatant was removed, the wells were washed 3 times with PBS, and fresh-growth cardiac fibroblast media was added. To isolate CMs, the supernatant was seeded on standard polystyrene cell culture plates coated with 0.1% gelatin (Sigma-Aldrich, G1890) dissolved in PBS.

Adult mouse ventricular cardiac fibroblast isolation

8 to 12-week-old male C57BL/6J mice (male) were anesthetized with an intraperitoneal injection of 0.4 mL of ketamine-xylazine cocktail (13 mg/mL ketamine and 0.88 mg/mL xylazine in PBS) and sacrificed by cervical dislocation. The thoracic cavity was opened, and the hearts were removed and placed in a petri dish containing PBS. The heart was squeezed gently with a tweezer to remove blood from the chambers, and the atria, vessels, and external connective tissue were removed. The ventricles were minced into 1-2 mm pieces with scissors and placed in scintillation vials containing a magnetic stir bar. Enzyme mix from the Neonatal Heart Dissociation Kit (Miltenyi Biotec, 130-098-373) was added according to the manufacturer's instructions to the scintillation vials, and the hearts were incubated at 37℃ for 45 minutes on a stir plate. The digestion was stopped by adding normal-growth cardiac fibroblast media (DMEM with 10% FBS, 1% penicillin-streptomycin, and 1% L-glutamine). The digested heart solutions were filtered through a 70μm strainer, centrifuged, and the cell pellet was resuspended in normal growth cardiac fibroblast media. Cells were seeded on standard polystyrene cell culture plates for 2 hours, during which adherent cells would attach. The supernatant containing cardiomyocytes was removed, centrifuged, and immediately lysed in the RNA extraction buffer TRIzol and stored at -80℃ before RNA isolation. Fibroblasts adhered to polystyrene plates were washed 3 times with PBS, and fresh growth cardiac fibroblast media was added.

Cell culture

Neonatal/adult cardiac fibroblasts and neonatal cardiomyocytes

After digestion of tissue and extraction of the supernatant, neonatal/adult cardiac fibroblasts and neonatal cardiomyocytes were cultured in cardiac fibroblast media (DMEM with 10% FBS, 1% penicillin-streptomycin, and 1% L-glutamine) at 37℃ and 5% CO_2 for 20 hours overnight. After 20 hours of culture, neonatal cardiomyocytes were lysed by adding TRIzol to wells and stored at -80℃ before RNA isolation. To induce myofibroblast differentiation in both neonatal and adult cardiac

fibroblasts, cells were treated with low-serum cardiac fibroblast media (DMEM with 1% FBS, 1% penicillin-streptomycin, and 1% L-glutamine) containing 10ng/mL human cell-expressed TGFβ1 (R&D Systems, 7754-BH-025/CF) and 1μM angiotensin II (Sigma-Aldrich, A9525) or vehicle (4mM HCl and water). Cardiac fibroblasts were treated for 48 hours (with an additional treatment following 24 hours) before lysing cells with TRIzol and storing at -80℃ before RNA isolation.

Overexpression of Slit2 in neonatal cardiac fibroblasts

Adenovirus to express eGFP was purchased from Vector Biolabs (#1060, viral titer 1.0x10^10 PFU/mL). Adenovirus to express □-galactosidase was purchased from Vector Biolabs (#1080, viral titer 1.0x10^10). Adenovirus to express human Slit2, with an HA tag, was purchased from Applied Biological Materials (443540951-HA, viral titer 1.0x10^6 PFU/mL). Both Adenoviruses contained the human adenovirus type5 (dE1/E3), and transgene expression was controlled by the CMV promoter. To ensure efficient expression of transgenes in cardiac fibroblasts, 2μL/mL of Ad-eGFP, 2μL/mL of Ad-βGal, or 10μL/mL of Ad-Slit2 in low-serum media were required for 24 hours. Following incubation with viruses, cells were treated with a cocktail of TGFβ1 and angiotensin II, as described above, for 48 hours (with an additional treatment following 24 hours) before lysing cells, conditioned media was removed from fibroblasts, and cells were then placed in TRIzol and stored at -80℃ before RNA isolation.

Myocardial infarction surgery

Mice were anesthetized by exposure to a 2% isoflurane/oxygen mixture and were administered Buprenorphine (2.5mg/kg) subcutaneously. A midline cervical incision was made to expose the trachea for intubation with a PE90 plastic catheter. The catheter was connected to a Harvard minivent supplying supplemental oxygen with a tidal volume of 225- 250 uL and a respiratory rate of 130 strokes/min

(Harvard Apparatus Model 845). Surgical plane anesthesia was subsequently maintained at 1-1.5% isoflurane. The skin was incised, and the chest cavity opened at the level of the 4th intercostal space. The mouse was placed on a heating pad (half-inch plexiglass between the animal and the heating pad). Oral intubation was employed by placing PE 90 tubing in the mouth and advancing slowly into the trachea. Mechanical P.I. ventilation began with a tidal volume of approximately 0.4 ml at 130 breaths/min. Maintenance anesthesia was kept at 1.25 - 1.75% isoflurane. After intubation, a midline incision was made between the sternum and the left internal mammalian artery. Alternatively, a lateral incision was made in the fourth intercostal space. The heart was exposed, and the left coronary artery was ligated intramurally 2mm from the origin with an 8-0 proline suture. Lungs were reinflated, and the chest was closed in two layers; the ribs (inner layer) were closed with 6-0 coated vicryl sutures in an interrupted pattern. The skin was closed using 6-0 nylon or silk sutures in a subcuticular manner. The anesthesia was stopped, and once the mouse was breathing independently, it was removed from the ventilator and allowed to recover in a clean cage on a heated pad. Sham surgeries were performed identically to MI procedures without an attempt of ligation of the left anterior artery.

Echocardiography

Echocardiography was performed using the Vevo 3100 (Fuji Film Visual Sonics) at baseline and longitudinally to evaluate cardiac function and recovery from surgical procedures. Briefly, animals were lightly anesthetized to ensure similar heart rates across animals and to allow for comparison between different time points. Hair was removed from the chest, and the mouse was gently restrained and placed on a micromanipulator platform following electrophysiologic and myocardial structure-function analysis. Vevo LAB software (Fuji Film Visual Sonics) was used for cardiac function and physiological measurements based on the long-axis view of the heart.

Adeno-associated virus serotype 9 (AAV9) delivery to mice via retro-orbital injection

Adeno-associated viruses serotype 9 (AAV9) were generated by Vector Builder to express Slit2 tagged with eGFP or to express eGFP alone using the cardiac troponin T (cTNT) promoter. To create recombinant AAV, the transfer plasmid carrying the gene of interest is co-transfected with a Rep-cap plasmid and helper plasmid encoding adenovirus genes (E4, E2A, and VA) that mediate AAV replication into HEK293T packaging cells. After a short incubation period, viral particles are harvested from cell lysate or supernatant, depending on serotype, and concentrated by PEG precipitation. For ultra-purified AAV (in vivo grade), viral particles are further purified and concentrated by cesium chloride (CsCl) gradient ultracentrifugation. AAV titer is measured using a qPCR-based approach. Viruses are formulated in PBS buffer (pH 7.4) supplemented with 200mM NaCl and 0.001% pluronic F-68. For our proposed experiment, ultra-purified recombinant AAV9 virus was made from the following plasmids: pAAV[Exp]-cTnT>mSlit2[NM_001291227.2](ns):T2A:EGFP:WPRE (Vector ID: VB220308-1230ngp) and pAAV[Exp]-cTnT>EGFP:WPRE (Vector ID: VB220216-1347cjv). Delivery of AAV9 at 3.00 x 10^{10} viral genomes/gram weight of mouse was prepared in sterile PBS and up to 100μL per injection, utilizing 25-gauge insulin needles. For this experiment, C56BL/6J female mice were purchased directly from the Jackson Laboratory at 5 weeks of age and treated with AAV9 two weeks later. To begin, mice were placed into an inhalational chamber filled with an isoflurane/oxygen mixture. Once the animal was completely anesthetized, the system was switched to flow through a nose cone, and the animal was repositioned in the nose cone. The skin above the eye was drawn back, and the injection needle was inserted, with a bevel facing the midline at an angle of approximately 30° into the medial canthus and through the conjunctival membrane[1]. The needle was positioned behind the globe of the eye in the retro-orbital sinus before the injectate was released. Following injection, the mouse was monitored on a heating pad before returning to the cage. Cardiac transgene transduction was validated by RT-qPCR and in situ hybridization experiments. Two weeks after AAV9 delivery, myocardial infarction surgeries were performed on mice as described above.

Adult heart tissue isolations

Hearts were harvested from adult mice following treatment with control or Slit2 AAV9 at 7, 14, and 28 days post-surgery in an unbiased manner. Sham hearts were isolated at 28 days post-surgery. Mice were sacrificed with ketamine/xylazine sedation followed by cervical dislocation, and excision of hearts was performed on Sham hearts and MI hearts at 7 and 14 days and immediately placed in PBS to remove excess blood. To allow for isolation of tissue for RNA isolations and immunohistochemical analysis from these groups, the left ventricle was cut sagitally, one half was immediately submerged in 10% neutral buffered formalin, and the other half of the tissue was flash frozen in liquid nitrogen and underwent cryo-pulverization using a CellCrusher-Mini, then lysed in TRIzol and stored at -80℃ before RNA isolation. Whole hearts harvested at 28 days post-MI were arrested in diastole by perfusing hearts with 0.1 M Cadmium Chloride (Sigma-Aldrich, 655198)/Potassium Chloride (Sigma-Aldrich, P3911), followed by perfusion with PBS for 5 minutes, then 10% neutral buffered formalin for 10 minutes at 80–100 mmHg via retrograde cannulation of the abdominal aorta. Retroperfused hearts were removed from the thoracic cavity and placed in 10% neutral buffered formalin for 16-24 hours before submission for processing.

Immunofluorescence staining (Paraffin)

Hearts were harvested and fixed in 10% neutral buffered formalin in PBS for 16-24 hours at room temperature. After fixation, tissues were dehydrated in 70% ethanol before embedding in paraffin wax (performed by UCLA TPCL) and sectioned at 5μm thickness. To perform immunofluorescence on paraffin-embedded sections, slides were deparaffinized in 3 changes of xylene (5 minutes each), followed by 3-minute incubations in 100% ethanol (EtOH, 3x), 95% EtOH (2x), 70%EtOH (1x) and then placed in distilled water. Specifically, antigen retrieval was performed in Antigen Retrieval pH6 Buffer (Akoya Biosciences, AR600), followed by quenching in 3% H_2O_2 (Sigma-Aldrich, H1009) in 15mM NaCl/100mM Tris pH 7.5 (TN buffer). Slides were then blocked 1X Antibody Diluent/Block (Akoya Biosciences, ARD1001). Primary antibodies were incubated overnight at 4°C at validated dilutions in

Antibody Diluent/Block. After overnight incubation (16-18 hours), slides were washed in 1X TN buffer, followed by secondary antibody incubation for two hours at room temperature in Antibody Diluent/Block. Amplification was performed as necessary (Biotin-Streptavidin or HRP-OPAL Dye). Slides were washed with 1X TN following secondary incubation with the final wash containing 4',6-Diamidino-2-Phenylindole, Dihydrochloride (DAPI, Fisher Scientific, D3571) for at least 10 minutes to stain for nuclei. Slides were mounted with ProLong Gold Antifade Mountant (Thermo Fisher Scientific, P36934) before being imaged on a Zeiss 700 or 880 Confocal Microscope. Antibodies and dilutions for immunofluorescence are listed in **Table 1.**

Assessment of vessel density using Isolectin GS-IB$_4$ staining

To quantify vascular density, tissue sections were processed and stained with isolectin and cardiac troponin T **(Table 1)** as described above. Z-stack images were taken for each heart, and quantitative analysis represented the average counting of at least 3 fields of view. Vessel count per cubic micron was quantified using Image J Version 2.0. software (National Institutes of Health, USA). Data was represented as the total number of IB4$^+$ vessels divided by the total number of DAPI$^+$ nuclei.

Assessment of proliferation using BrdU and ERG labeling

To label proliferating cells in the heart, mice were given an intraperitoneal injection of Bromodeoxyuridine (BrdU, Sigma Aldrich, 19-160) at 50mg/kg in sterile saline every 3 days. Tissue sections were processed for immunofluorescence staining to detect BrdU and ERG **(Table 1)** using the above protocol. BrdU incorporation was measured using Imaris Software (Oxford Instruments), and quantitative analysis represented the counting of at least 3 fields of view of the border zone region from biologically independent samples per group. The total number of DAPI$^+$ nuclei was counted to quantify proliferating cells in hearts, followed by the total BrdU$^+$ and ERG$^+$, and lastly, the cells co-labeled as BrdU$^+$/ERG$^+$. Data was represented as the total number of positive or double-positive cells divided by the number of nuclei.

Assessment of cardiomyocyte cross-sectional in vivo

Tissue sections were processed for immunofluorescence staining to detect cardiac troponin T and WGA **(Table 1)** as described above. Cardiomyocyte cross-sectional area (CSA) was measured using Image J Version 2.0. software (National Institutes of Health, USA), and quantitative analysis was represented by counting at least 3 fields of view of the border zone region from biologically independent samples per group.

Picrosirius red staining

Formalin-fixed paraffin-embedded (FFPE) tissue sections were deparaffinized and hydrated to DI water as follows: 3 changes of xylenes, 3 changes of 100% Ethanol, 2 changes of 95% Ethanol, 1 change of 70% Ethanol, followed by 2 changes of DI water. Slides were stained using the Picrosirius Red Stain Kit (Polysciences, 24901). Picrosirius red stain was added to the slides for 20 minutes without disturbance. Following the 20-minute incubation, Picrosirius red stain was rinsed with hydrochloric acid for 1 minute twice. Following the second hydrochloric acid incubation and rinse, slides were placed in 70% Histology-grade ethanol for 3 minutes, followed by slides in 100% Histology-grade ethanol for 3 minutes. Following ethanol incubation, slides were set to dry for approximately 30 seconds before applying Permount and a cover slip. Stained slides were imaged by bright field imaging, and tile scanned at a magnification of 20x using the Applied Imaging Leica Aperio Verso scanner. Quantitation of Picrosirius was performed using Image J Version 2.0. software (National Institutes of Health, USA) by normalizing the area of the Picro Sirius stain to the size of the left ventricular free wall.

In situ hybridization assays

Tissue sections were harvested and fixed in 10% neutral buffered formalin in DPBS for 18-24 Hours at room temperature on a rocking platform. After fixation, tissue was dehydrated in an ethanol series followed by xylene before embedding tissue in paraffin wax and cutting hearts into 5μm sections

using a microtome. After sectioning, slides were allowed to dry overnight at room temperature and stored with desiccants for long-term storage. We utilized the RNAscope Multiplex Fluorescent V2 Assay (Advanced Cell Diagnostics, 323100) for in situ hybridization assays per the manufacturer's instructions for formalin-fixed paraffin-embedded (FFPE) tissue. DAPI was added to sections after the last wash step for 1 minute. Slides were mounted with ProLong Gold Antifade Mountant (Thermo Fisher Scientific, P36934) before being imaged on a Zeiss 700 or 880 Confocal Microscope. RNA probes and secondary detection methods are listed in **Table 2**.

In situ cell death detection

To detect apoptotic cells following myocardial infarction, sections of formalin-fixed and paraffin-embedded heart tissue were subjected to an in-situ cell death detection kit, fluorescein (Millipore Sigma). Briefly, tissue sections were deparaffinized and subjected to a permeabilization solution (0.1% Triton-X-100, 0.1% sodium citrate) for 15 minutes. TUNEL reaction mixture was created and placed on slides for 2 hours at 37°C in a humidified atmosphere in the dark. Slides were washed twice with 1x TN and incubated with DAPI before coverslipping and imaging using confocal microscopy.

Isolation of neonatal rat ventricular cardiomyocytes (NRVMs)

Neonatal rat pups (aged postnatal day 2-3) purchased from Charles River were euthanized by hypothermia and decapitation, and atria were removed from excised hearts. Ventricles were then briefly rinsed in ice-cold 1x ADS buffer (116mM NaCl, 18mM HEPES, 845μM NaHPO4, 5.55mM Glucose, 5.37mM KCl, 831μM MgSO4, 0.002% Phenol Red, pH 7.35±0.5) and minced via ~200 cuts with sterile micro scissors. Minced ventricles were then subjected to 5-10 serial digestions in a Wheaton 356945 Celstir 50mL Jacketed Glass Spinner Flask with Double Sidearms at room temperature using 300μL/heart of ADS buffer containing 0.1% Trypsin (Sigma-Aldrich, T4799) and

0.002% DNase (Worthington Biochemical Corp., LS002006). Each digestion lasted 10-20 minutes. The cell suspension from the first digestion was discarded as it contained unwanted dead and red blood cells. The cell suspension from each subsequent digestion was then combined with fetal calf serum to a concentration of 50%. The pooled cell suspensions were then filtered with a 100µm nylon cell strainer (Foxx Life Sciences, 4100003-OEM) and centrifuged for 8 minutes at 1500xg. The resulting cell pellet was then resuspended in 8mL of 1×ADS buffer. Stock Percoll was prepared by combining 9 parts of Percoll (GE Healthcare, 17-0891-02) with 1 part of clear (without phenol red) 10×ADS. The stock Percoll was used to make the Percoll for the top (density= 1.059 g/ml; 1 part Percoll stock added to 1.2 parts 1x ADS without phenol red) and bottom (density= 1.082 g/ml; 1 part Percoll stock added to 0.54 parts 1x ADS with phenol red) layers. The gradient, consisting of 4 ml top Percoll and 3 ml bottom Percoll, was set in a 15 ml conical tube by pipetting the top Percoll first and layering the bottom Percoll gently underneath. The cells (in 2 ml red 1x ADS buffer) were layered on the top of the discontinuous Percoll gradient (4 gradients in total) and centrifuged at 1500xg for 30 minutes at 4oC, with no deceleration break to separate the myocytes from non-myocytes. The myocytes, concentrated between the two Percoll layers as well as myocytes that centrifuged to the bottom of the tube, were then collected, washed once with 1x ADS buffer, and resuspended in a plating medium composed of DMEM/F:12 (ThermoFisher Scientific, 11330-32) supplemented with 10% FCS and penicillin/streptomycin.

Treatment of NRVMs with adenoviruses and phenylephrine

NRVMs were serum-starved for 24 hours in serum-free DMEM/F:12 supplemented with penicillin/streptomycin and insulin-transferrin-selenium (ITS, Thermo Fisher Scientific, 41400045) and then treated with adenoviruses described in a previous section, with the following multiplicity of infection (MOI): Adeno-GFP MOI: 10, and Adeno-SLIT2 MOI: 0.15) dissolved in DMEM/F:12 supplemented with ITS and antibiotics for 24 hours. NRVMs were washed and treated with 50µM phenylephrine (Sigma-Aldrich, P6126) dissolved in DMEM/F:12 supplemented with ITS and

antibiotics for 24 hours before RNA or immunocytochemical protocols described below.

Immunocytochemical assessment of NRVMS, HCAECs and cFBs

NRVMs were plated at 350,000 cells/well on fibronectin-coated glass slides (Lab-Tek II). HCAECS and cardiac fibroblasts were seeded at 100,000 cells/well on fibronectin-coated 24-well glass-bottom plates. After adenovirus incubation and treatments as described above, slides or wells were washed two times with 0.5 mL/well ice-cold DPBS. Slides were then fixed with 4% PFA in PBS on ice for 15 min. Slides were then washed three times with ice-cold PBS (5 min/wash), followed by permeabilization with 0.5% Triton X-100, 3 mm EDTA for 10 min on ice, and then washed three times with PBS (5 min/wash) and blocked for 1hr with 10% donkey serum in PBS. Slides were then incubated with primary antibodies diluted in 10% donkey serum for 16h at 4°C. Slides were washed six times with ice-cold PBS (5 min/wash) and then incubated at room temperature, in the dark for 90 min, with the fluorophore-conjugated secondary antibodies diluted in 10% donkey serum. Slides were washed six times with ice-cold PBS (5 min/wash). Nuclei were counterstained for 5 min with DAPI (Fisher Scientific, BDB564907). Slides were mounted with ProLong Gold Antifade Mountant (Thermo Fisher Scientific, P36934) before imaging on a Zeiss 700 or 880 Confocal Microscope. Antibodies and dilutions for immunocytochemistry are listed in **Table 1.** NRVM CSA following treatment with adenoviruses and phenylephrine involved tracing cardiac troponin T labeled cardiomyocytes using Image J Version 2.0. software (National Institutes of Health, USA).

Human Coronary Artery Endothelial Cells (HCAEC)

Human Coronary Artery Endothelial Cells (HCAECs) were purchased from Lonza (CC-2585). HCAECs were seeded at a density of 40,000 – 80,000 cells per well on 24-well plates using EBM-2 Endothelial Cell Growth Basal Medium-2 (Lonza, CC-3156) supplemented with 5% fetal bovine serum (Lonza Walkersville EGM-2 MV Microvascular Endothelial SingleQuots Kit, CC-4147). Before each experiment, cells were placed in EBM-2 medium with 1% fetal bovine serum for 16 hours to serum-starve cells. Following serum starvation, non-adherent cells and debris were aspirated, and HCAECs

were washed with phosphate-buffered saline (PBS) before treatment with cardiac fibroblast conditioned media.

Proteome Profiler Mouse XL Cytokine Array (Biotechne R&D Systems)

Conditioned media from cardiac fibroblasts infected with adenoviruses (AdenoControl/AdenoSlit2) and treated with vehicle (4mM HCl and water) or TGFβ1 (R&D Systems, 7754-BH-025/CF) were collected and pooled from multiple cell culture plate wells based on treatment group. Conditioned media were centrifuged at 1,000 rpm for 5 minutes to pellet non-adherent cells and debris. Conditioned media was then profiled using Biotechne R&D Systems (Catalog #: ARY028) Proteome Profiler Mouse XL Cytokine Array according to the manufacturer's instructions. Membranes were developed and imaged using a Bio-Rad Chemidoc Touch Screen Imaging System (Bio-Rad). Uncropped exposed membranes are included in the supplemental documents.

RNA isolation, cDNA synthesis, and Real-Time Quantitative Reverse Transcription PCR

To perform gene expression analyses, isolated cells were sorted directly into TRIzol. RNA was extracted from cells or whole heart samples following the manufacturer's protocol. Extracted RNA was quantified using a NanoDrop Spectrophotometer (Thermo Fisher Scientific) and then converted into complementary DNA (cDNA) using Verso cDNA Synthesis Kit (Thermo Fisher Scientific, AB1453A) following the manufacturer's instructions. RT-qPCR reactions were prepared with SsoAdvanced Universal SYBR Green Supermix (Bio-Rad, 1725274), and specific primers designed for each target gene are listed in **Table 3**. The reactions were run on a CFX Opus Real-Time PCR (Bio-Rad). Data analysis was performed using the ΔΔCt method, and gene expression levels were normalized to *18s* for mice and humans and *Actb* for detection in rats.

Analysis of Single Nuclei RNA-sequencing

Count matrices were retrieved from the ArrayExpress database of the European Bioinformatics Institute and integrated using the Seurat R package[2]. Quality control, identification of variable genes, principal

component analysis (PCA), and non-linear reduction using uniform manifold approximation and projection (UMAP) were performed using Seurat (v5.1). Data was visualized in UMAP space, and clusters were defined using Enrichr and PanglaoDB. Seurat was used to visualize differentially expressed genes across all cardiac cell clusters.

Analysis of Single-cell RNA Sequencing Datasets

Expression levels of the genes of interest in cardiac cell types were downloaded from the CLARA and CardiacFibroAtlas web portals. Each dataset was processed as previously described in each study[3,4]. Publicly available single-cell RNA sequencing datasets from human and mouse cardiovascular studies were integrated. Raw data was processed with the Cell Ranger pipeline and analyzed using the Seurat R package for quality control, clustering, and dimensionality reduction. Differential expression analysis was performed using the MAST method to identify key genes across experimental conditions.

Statistics

Data were presented as mean +/− standard error of the mean (SEM) for all bar graph data. Statistical analyses were performed using unpaired two-tailed Student’s t-test when comparing two groups and One-Way and Two-Way ANOVA when comparing multiple groups. Tukey post-test was used to correct multiple comparisons made by One-Way ANOVA, and Sidak post-test was used to correct multiple comparisons made by Two-Way ANOVA. All measurements in this paper were acquired from different biological samples, and no samples were measured repeatedly. Bar graph data analysis Graph Pad Prism 8 for macOS (Version 10.3.1). A p-value less than 0.05 was considered statistically significant. For figure presentation, only p-values below the threshold of $p < 0.15$ were displayed.

Data availability

The raw and processed single-nuclei RNA-sequencing data presented in Supplementary Figure 7 is available in the ArrayExpress database of the European Bioinformatics Institute and may be found through accession number E-MTAB-7869.

Code availability

All analyses were performed using standard protocols with previously described R packages. The R scripts are available upon request.

Supplemental Table 1. Antibodies/Stains for Immunocyto- and histochemistry

	Supplementary Table 1. Antibodies/Stains used for immunocyto- and histochemistry							
	**Primary Antibody**	**Manufacturer**	**Catalog No.**	**Dilution**	**Secondary Antibody**	**Manufacturer**	**Catalog No.**	**Dilution**
**1**	DAPI (nuclear dye)	Fisher Scientific	D3571	1:10,000	NA	NA	NA	NA
**2**	goat anti-ERG	Abcam	ab115555	1:100	donkey anti-goat Cy3	Jackson ImmunoResearch	705-165-147	1:100
**3**	goat anti-GFP	Rockland	600-101-215S	1:100	donkey anti-goat 488	Jackson ImmunoResearch	705-545-147	1:100
**4**	goat anti-PDGFRα	R&D Systems	AF1062	1:100	donkey anti-goat HRP	Jackson ImmunoResearch	705-035-147	1:2,000
**5**	Isolectin-647 (vascular dye)	Fisher Scientific	I32450	1:100	NA	NA	NA	NA
**6**	mouse anti-cTNT	Thermo Fisher Scientific	MA5-12960	1:100	donkey anti-mouse 647/488	Jackson ImmunoResearch	715-605-150/715-545-150	1:100
**7**	mouse anti-Robo1	Proteintech	67922-1-IG	1:100	donkey anti-mouse 647/488	Jackson ImmunoResearch	715-605-150/715-545-150	1:100
**8**	Phalloidin 647	Fisher Scientific	A22287	1:400	NA	NA	NA	NA
**9**	rabbit anti-ERG	Abcam	ab92513	1:100	donkey anti-rabbit Biotin	Jackson ImmunoResearch	711-065-152	1:5,000
**10**	rabbit anti-Slit2	Fisher Scientific	50-173-2773	1:100	donkey anti-rabbit Cy3	Jackson ImmunoResearch	711-165-152	1:100
**11**	rat anti-BrdU	Abcam	ab6326	1:100	donkey anti-rat HRP	Jackson ImmunoResearch	712-035-153	1:2,000
**12**	rat anti-Ki67	Thermo Fisher Scientific	14-5698-82	1:50	donkey-anti-rat 488	Jackson ImmunoResearch	712-545-153	1:100
**13**	WGA-647 (cell membrane dye)	Fisher Scientific	W32466	1:100	NA	NA	NA	NA
**14**	rabbit anti-VEGFA	**Abcam**	**ab52917**	1:100	donkey anti-rabbit Cy3	Jackson ImmunoResearch	711-165-152	1:100
**15**	rabbit anti-FGF9	Abcam	ab206408	1:100	donkey anti-rabbit Cy3	Jackson ImmunoResearch	711-165-152	1:100
**16**	rabbit anti-VEGFR2 (Tyr1175)	Cell Signaling Technology	2478	1:100	donkey anti-rabbit 488	Jackson ImmunoResearch	711-545-152	1:100
**17**	rabbit anti-Akt (Ser473)	Cell Signaling Technology	4060	1:100	donkey anti-rabbit 488	Jackson ImmunoResearch	711-545-152	1:100
**18**	rabbit anti-CD68	Abcam	ab125212	1:100	donkey anti-rabbit 488	Jackson ImmunoResearch	711-545-152	1:100
**19**	rabbit anti-Fibrinogen	Abcam	ab92572	1:100	donkey anti-rabbit 488	Jackson ImmunoResearch	711-545-152	1:100
	**Amplified Y/N**	**Tertiary Antibody**	**Manufacturer**	**Catalog No.**	**Dilution**	**Application**		
**1**	NA	NA	NA	NA	NA	IF - Paraffin/Cyto		
**2**	N	NA	NA	NA	NA	IF - Cyto		
**3**	N	N	NA	NA	NA	IF - Cyto		
**4**	Y	OPAL 570	Akoya Biosciences	FP1488001KT	1:100	IF - Paraffin		
**5**	NA	NA	NA	NA	NA	IF - Paraffin		
**6**	N	NA	NA	NA	NA	IF - Paraffin/Cyto		
**7**	N	NA	NA	NA	NA	IF - Paraffin/Cyto		
**8**	NA	NA	NA	NA	NA	IF - Cyto		
**9**	Y	Streptavidin 488	Jackson ImmunoResearch	016-540-084	1:100	IF - Paraffin		
**10**	N	N	NA	NA	NA	IF - Paraffin/Cyto		
**11**	Y	OPAL 570	Akoya Biosciences	FP1488001KT	1:100	IF - Paraffin		
**12**	N	NA	NA	NA	NA	IF - Cyto		
**13**	NA	NA	NA	NA	NA	IF - Paraffin		
**14**	N	NA	NA	NA	NA	IF - Cyto		
**15**	N	NA	NA	NA	NA	IF - Cyto		
**16**	N	NA	NA	NA	NA	IF - Cyto		
**17**	N	NA	NA	NA	NA	IF - Cyto		
**18**	N	NA	NA	NA	NA	IF - Paraffin		
**19**	N	NA	NA	NA	NA	IF - Paraffin		

Supplemental Table 2. RNAscope mRNA Probes for In Situ Hybridization

**Supplementary Table 2. RNAscope Probes for In Situ Hybridizations**		
Gene Target	RNAscope Catalog #	Akoya Biosciences # for Fluorescent Detection & Dilution
SLIT2	4499691-C2	Akoya Biosciences OPAL 570 (FP1488001KT); 1:750
TCF21	508661-C1	Akoya Biosciences OPAL 520 (FP1487001KT); 1:750
WT1	432711-C1	Akoya Biosciences OPAL 520 (FP1487001KT); 1:750
ROBO1	475951-C3	Akoya Biosciences OPAL 690 (FP1497001KT); 1:750

Supplemental Table 3. Primer sequences for quantitative RT-qPCR

**Supplementary Table 3. Primer Sequences for RT-qPCR**			
Species	Gene Target	Forward Primer	Reverse Primer
mus musculus/homo sapien	18S	CAT GGC CTC AGT TCC GAA AA	CGA GCC GCC TGG ATA CC
mus musculus	ACTA2	GTT CAG TGG TGC CTC TGT CA	ACT GGG ACG ACA TGG AAA AG
rat norvegicus	ACTB	CTTCCTTCCTGGGTATGGAATC	CTGTGTTGGCATAGAGGTCTT
mus musculus	AKT1	GTGACAACTCAGGGGCTGAA	CTCGTTCATGGTCACACGGT
mus musculus	ANGPT1	CATCAGCTCAATCCTCAGC	GGGGGAGGTTGGACAGTAA
mus musculus	ANGPT2	ACAGAGGAGATCAAGGCCTACT	GCCATCTTCTCGGTGTTGGA
mus musculus	ANGPTL2	CAG GAG AGA AGA GGC TTT CAG T	GCA GTC CAA GCC ACC AGT AG
mus musculus	APLNR	CCTCCATGCTCTGCTGTGAT	GCCCGGAAGAATAACTGGCT
mus musculus	COL1A1	TAGGCCATTGTGTATGCAGC	ACATGTTCAGCTTTGTGGACC
mus musculus	COL3A1	TAGGACTGACCAAGGTGGCT	GGAACCTGGTTTCTTCTCACC
mus musculus	COL4A1	AGGTCGCCCTGGATTTAACG	CCAATGCCAGGTTCTCCCTTT
mus musculus	COL5A1	CCAGAATCACTTCTTGGCCC	GCATCCACATAGGAGAGCAGTT
homo sapiens	ELAM1	AAGTGTGACCCTGGCTTCAG	AAACCAGGCTTCCATGCTCA
mus musculus	FGF1	AGCTTTCTCCCAAGAGACCA	TCATGGCGTTTGTGTCCTAT
mus musculus	FGF9	AAAGGGGATTCTCAGGCGGA	TCCAGAATGCCGAAGCGG
mus musculus	FN	AGACCTGGGAAAAGCCCTACCAA	ACTGAAGCAGGTTTCCTCGGTTGT
mus musculus	ICAM1	CTCGAGAGTGGACCCAACTG	TTCTCAAAGCACAGCGGACT
homo sapiens	ICAM1	CCAGGAGACACTGCAGACAG	CTTCACTGTCACCTCGGTCC
mus musculus	IGFBP3	AACCTGCTCCAGGAAACATC	ACAACCTGGCTTTCCACACT
mus musculus	IL1B	GCCACCTTTTGACAGTGATGAG	TGATGTGCTGCTGCGAGATT
homo sapiens	IL-6	CCACCGGGAACGAAAGAGAA	GAGAAGGCAACTGGACCGAA
mus musculus	MYH6	GTGCCAAGAAGATGCACGAC	GGCAGAGTCGAACGTTTATGT
mus musculus	MYH7	GTGGCTCCGAGAAAGGAAG	GAGCCTTGGATTCTCAAACG
mus musculus	NOS3	ATCCAGTGCCCTGCTTCA	GCAGGGCAAGTTAGGATCAG
homo sapiens	NOS3	GACCCACTGGTGTCCTCTTG	CCCGAACACACAGAACCTGA
rat norvegicus	NPPA	GCGAAGGTCAAGCTGCTTCG	CTCTGGGCTCCAATCCTGTC
rat norvegicus	NPPB	GTGCTGCCCCAGATGATTCT	CAGCGGCGACAGATTAAGGA
mus musculus	PDGFA	GCTGCACTGGCTGTTGTAA	ACTTTGGCCACCTTGACACT
mus musculus	PECAM1	TGGTTGTCATTGGAGTGGTC	TTCTCGCTGTTGGAGTTCAG
mus musculus	POSTN	AAGCTGCGGCAAGACAAG	TCAAATCTGCAGCTTCAAGG
mus musculus	ROBO1	GCTGGCGACATGGGATCATA	AATGTGGCGGCTCTTGAACT
mus musculus	ROBO2	GCTGAGAATCGGGTGGGAAA	AACTGTGGAGGAGCAACAGG
mus musculus	ROBO4	ATGGAAATCTGGGCCTGGGA	GGAACCAGAGTTTGGACCTGGG
mus musculus	SLIT2	GGGTGGGCTTGTACACTCAG	TCTTCCTCATCACTGCAGACAA
homo sapiens	SLIT2	CTCCCACCTGAGAGGCCATA	CTGGTGACCACTGCAGACAA
mus musculus	SLIT3	GCGCGATTTGGAGATCCTCA	TGGAGTGTAGACGCAGAGTCC
mus musculus	TCF21	CATTCACCCAGTCAACCTGA	CCACTTCCTTCAGGTCATTCTC
homo sapiens	TIE2	GCCCTGCTGATACCAAATGC	AGGAACAGCACAGAAGCAGG
mus musculus	TNFA	ATGGCCTCCCTCTCATCAGT	TGGTTTGCTACGACGTGGG
mus musculus	VCAM1	CTCTTTATGTCAACGTTGCCCCC	TTTAGCTGTCTGCTCCACAGG
homo sapiens	VCAM1	GGACCACATCTACGCTGACA	TTGACTGTGATCGGCTTCCC
mus musculus	VEGFA	TGCGGATCAAACCTCACCAA	GGCTCACAGTGATTTTCTGGC
mus musculus	VEGFB	AGCCACCAGAAGAAAGTGGT	GCTGGGCACTAGTTGTTTGA
mus musculus	VEGFC	GAGCAGTTGCGGTCTGTGTC	GTCCCCTGTCCTGGTATTGAG

References

1. Challis RC, Ravindra Kumar S, Chan KY, Challis C, Beadle K, Jang MJ, Kim HM, Rajendran PS, Tompkins JD, Shivkumar K, et al. Systemic AAV vectors for widespread and targeted gene delivery in rodents. *Nat Protoc*. 2019;14:379-414. doi: 10.1038/s41596-018-0097-3
2. Vidal R, Wagner JUG, Braeuning C, Fischer C, Patrick R, Tombor L, Muhly-Reinholz M, John D, Kliem M, Conrad T, et al. Transcriptional heterogeneity of fibroblasts is a hallmark of the aging heart. *JCI Insight*. 2019;4. doi: 10.1172/jci.insight.131092
3. Dona MSI, Hsu I, Rathnayake TS, Farrugia GE, Gaynor TL, Kharbanda M, Skelly DA, Pinto AR. CLARA: A web portal for interactive exploration of the cardiovascular cellular landscape in health and disease. *bioRxiv*. 2021:2021.2007.2018.452862. doi: 10.1101/2021.07.18.452862
4. Patrick R, Janbandhu V, Tallapragada V, Tan SSM, McKinna EE, Contreras O, Ghazanfar S, Humphreys DT, Murray NJ, Tran YTH, et al. Integration mapping of cardiac fibroblast single-cell transcriptomes elucidates cellular principles of fibrosis in diverse pathologies. *Sci Adv*. 2024;10:eadk8501. doi: 10.1126/sciadv.adk8501

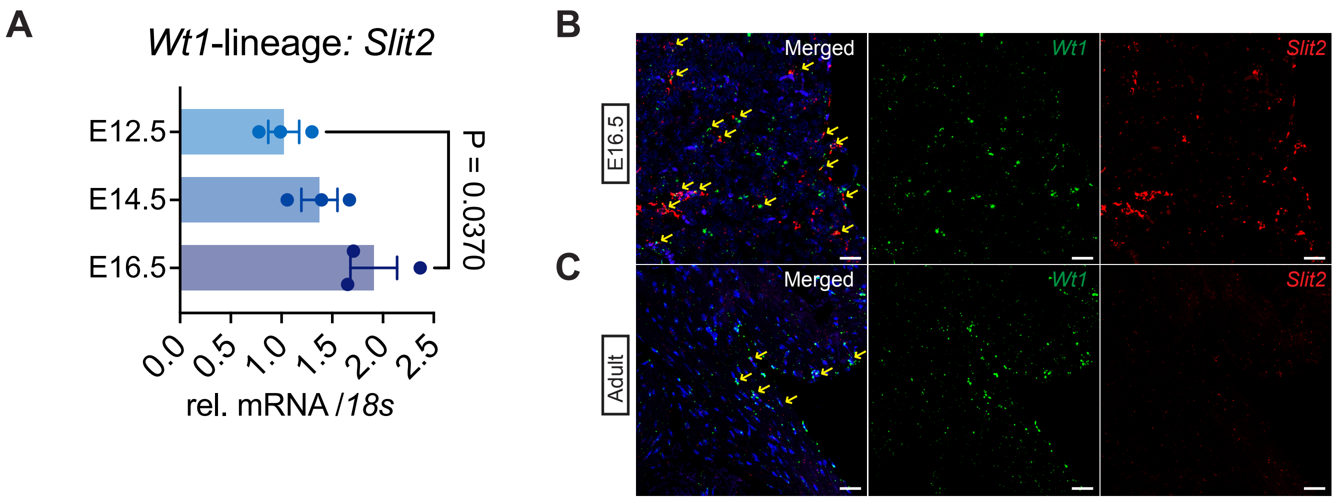

Supplemental Figure 1. Slit2 is enriched in epicardial-derived cells during development. (A) *Slit2* in *Wt1*-lineage epicardial cells isolated at embryonic (E) stages 12.5, 14.5, and 16.5 by fluorescence-activated cell sorting and quantified by RT-qPCR. N=3 per embryonic age. ONE-Way ANOVA determined P values. **(B)** *Slit2* (red), and *Wt1* (green) expression determined by fluorescence in situ hybridization in E16.5 and **(C)** 12-week-old adult hearts. DAPI staining was used to visualize nuclei (blue). Micrographs represent N=3 biological samples imaged at each age. Yellow arrows indicate co-localization of *Wt1*-expressing cells and Slit2. Scale bar, 20μm.

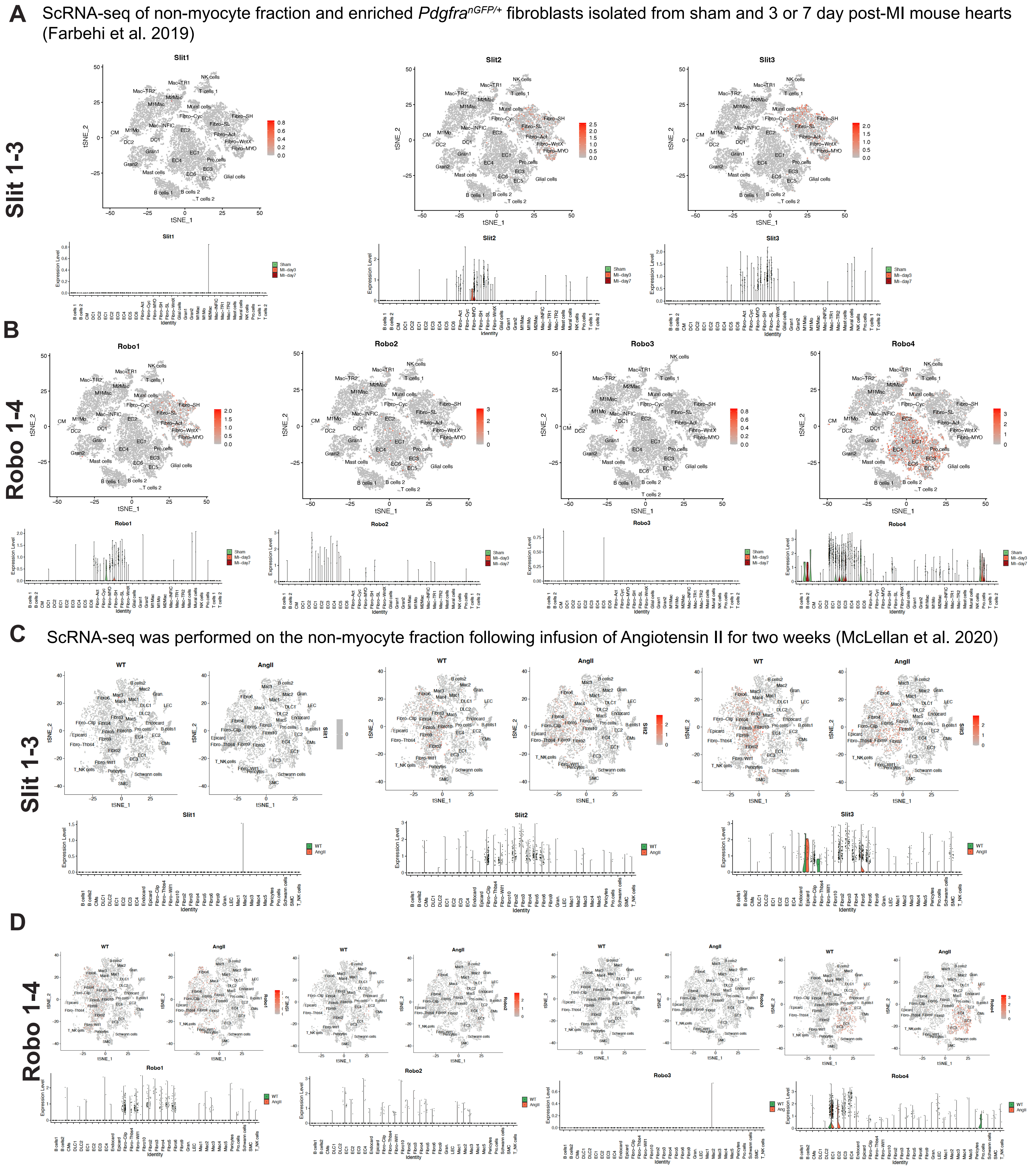

Supplemental Figure 2. Transcriptional analysis of the Slit2-Robo in cardiac cell types. (A-D) Single-cell RNA sequencing data obtained and visualized from CardiovascuLAR Atlas (CLARA). **(A, B)** Slit1-3 and Robo1-4 expression in non-cardiomyocytes and platelet-derived growth factor receptor alpha (PDGFRα) cells following sham surgery or 3 or 7 days of myocardial infarction. **(C, D)** Slit1-3 and Robo1-4 expression in non-cardiomyocytes following infusion of Angiotensin II to mice for 2 weeks. Data is represented as t-distributed stochastic neighbor embedding (t-SNE) and violin expression plots.

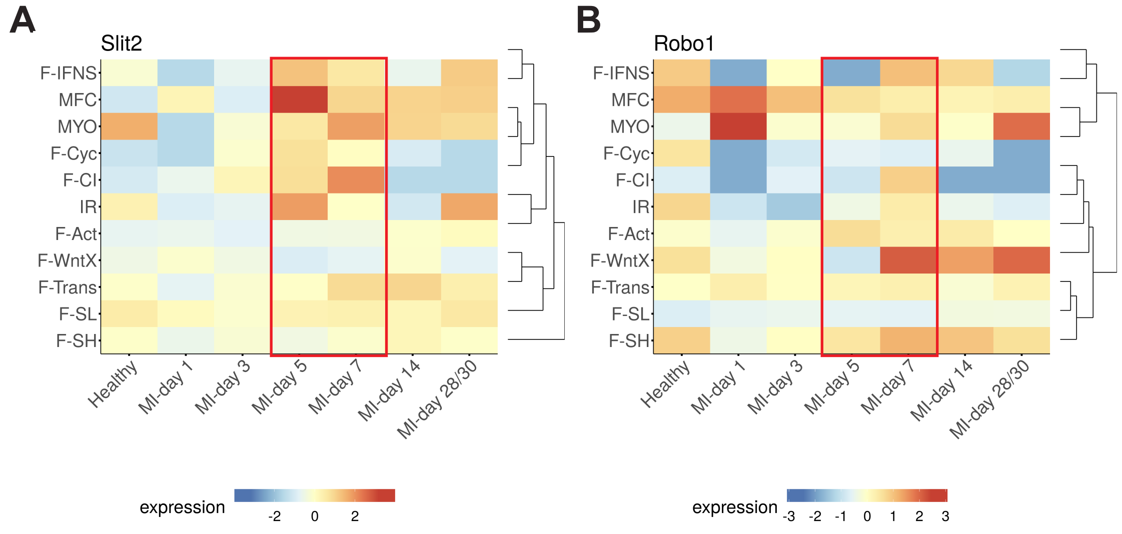

Cardiac Fibroblast Identities

F-Trans = Transitory
F-SL = Sca1-low
F-Act = Activated
F-SH = Sca1-high
MFC = Matrifibrocytes
MYO = Myofibroblasts
F-IFNS = IFN stimulated
F-WntX = Wnt-Expressing
F-Cl = Cycling Intermediate
IR = Injury Response
F-Cyc = Cycling

Supplemental Figure 3. Slit2-Robo1 transcriptional changes in cardiac fibroblasts during homeostasis and myocardial infarction. (A, B) Heatmap showing *Slit2* and *Robo1* across 11 cardiac fibroblast populations derived from healthy adult hearts and from hearts following different time points following myocardial infarction. Data and visualization obtained from CardiacFibroAtlas, a database integrating four single-cell RNA sequencing studies spanning seven time points of MI along with uninjured/sham controls (Farbehi et al. 2019, Forte et al. 2020, Janbandhu et al. 2022, Ruiz-Villalba et al. 2020).

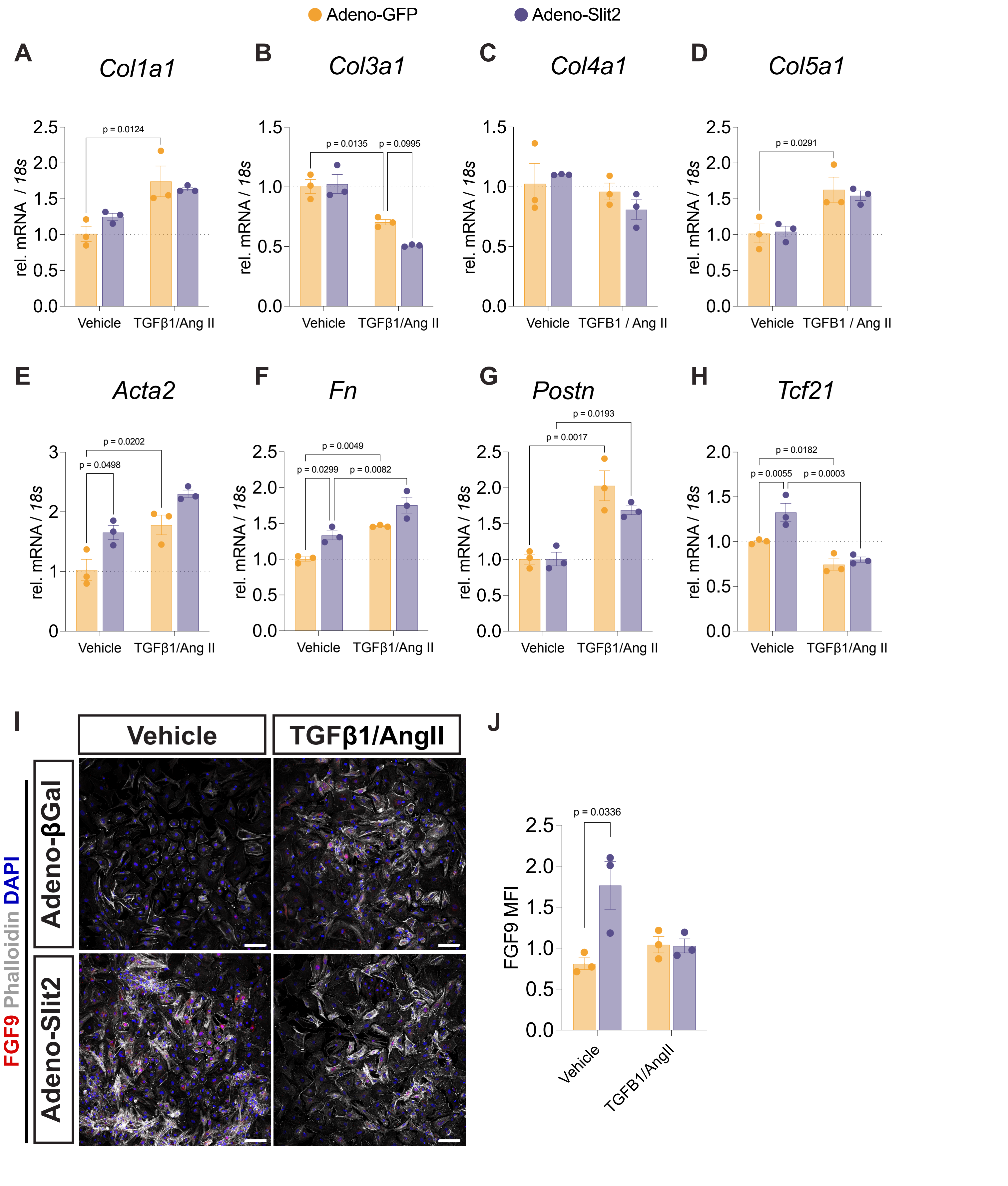

Supplemental Figure 4. SLIT2 Overexpression does not alter fibroblast phenotype or transition towards a myofibroblastic state. Expression of fibroblasts and extracellular matrix genes **(A-H)** following vehicle or TGFβ1/AngII treatment in AdenoGFP- or AdenoSLIT2-infected cFBs. N=3. Statistical comparisons were made using two-way ANOVA with Tukey's multiple comparisons test to determine P values. **(I-J)** Immunocytochemistry analysis of FGF9 protein expression in cFBs infected with either AdenoβGal or AdenoSLIT2 and treated with vehicle or TGFβ1/AngII. Representative immunofluorescence images **(I)** showing FGF9 (red), phalloidin (gray), and DAPI (blue). Quantification of FGF9 mean fluorescence intensity (MFI) is shown in **(J)**. N=3. Statistical comparisons was performed using unpaired student's t-test between conditions.

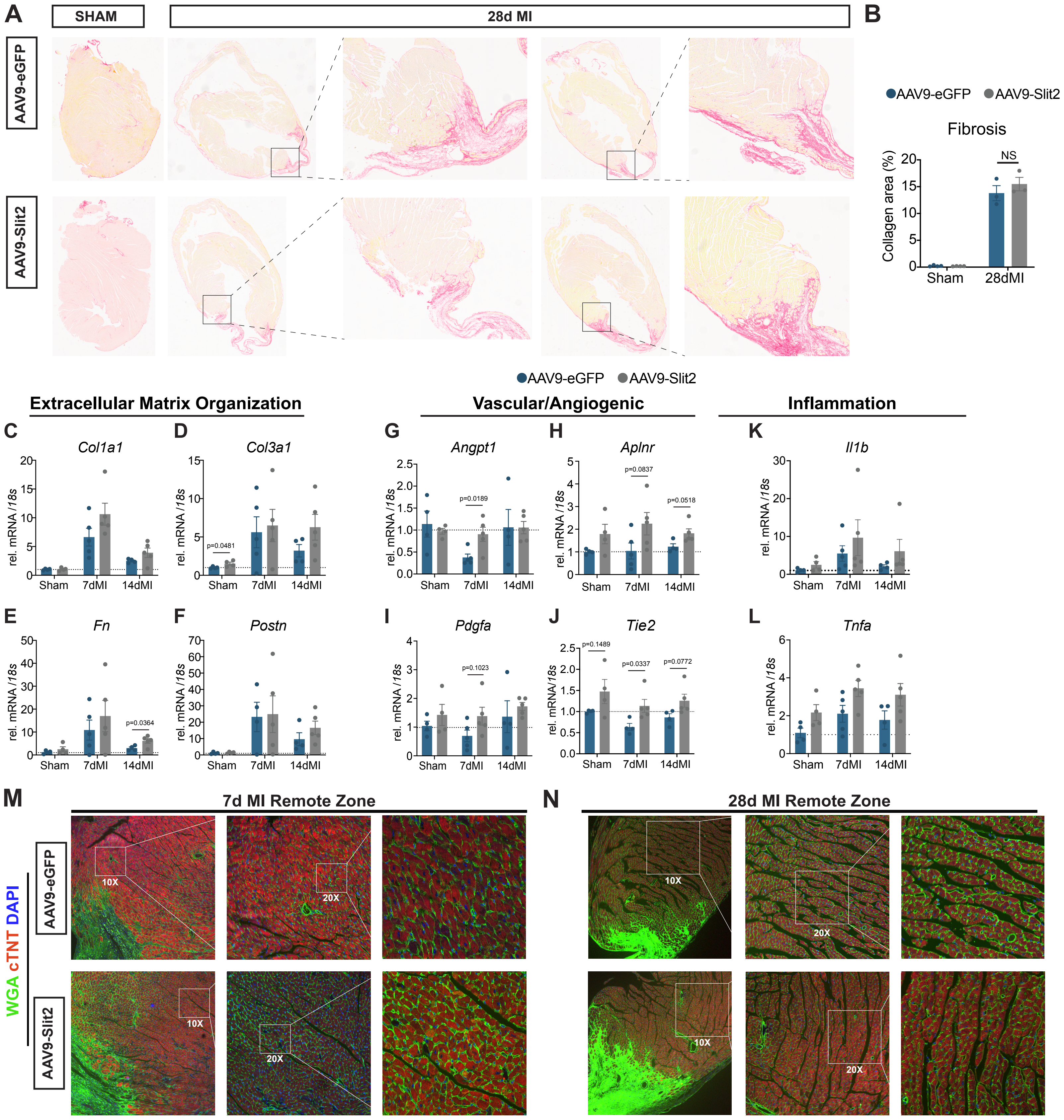

Supplemental Figure 5. Overexpression of Slit2 in the myocardium does not alter extracellular matrix deposition during myocardial infarction. (A) Representative Picrosirius Red-stained heart sections from AAV9-eGFP and AAV9-Slit2-treated mice subjected to sham surgery or 28 days of MI. **(B)** Quantification of collagen area in the left ventricular free wall between Sham/MI AAV9-eGFP (n=4/n=3) and AAV9-Slit2 (n=4/n=3) groups. **(C-F)** RT-qPCR analysis of extracellular matrix-related gene expression including *Col1a1*, *Col3a1*, *Fn*, and *Postn*. **(G-J)** Gene expression of vascular/angiogenic genes including *Angpt1*, *Aplnr*, *Pdgfa* and *Tie2*. **(K-L)** Gene expression of inflammation-associated genes including *Il1b* and *Tnfa*. Data presented as mean +/- SEM, n=4-5. **(M-N)** Representative images of wheat germ agglutinin (WGA) staining in the remote zone of hearts at 7 **(M)** and 28 **(N)** days post-MI. Insets show increasing magnification from 5X to 20X.

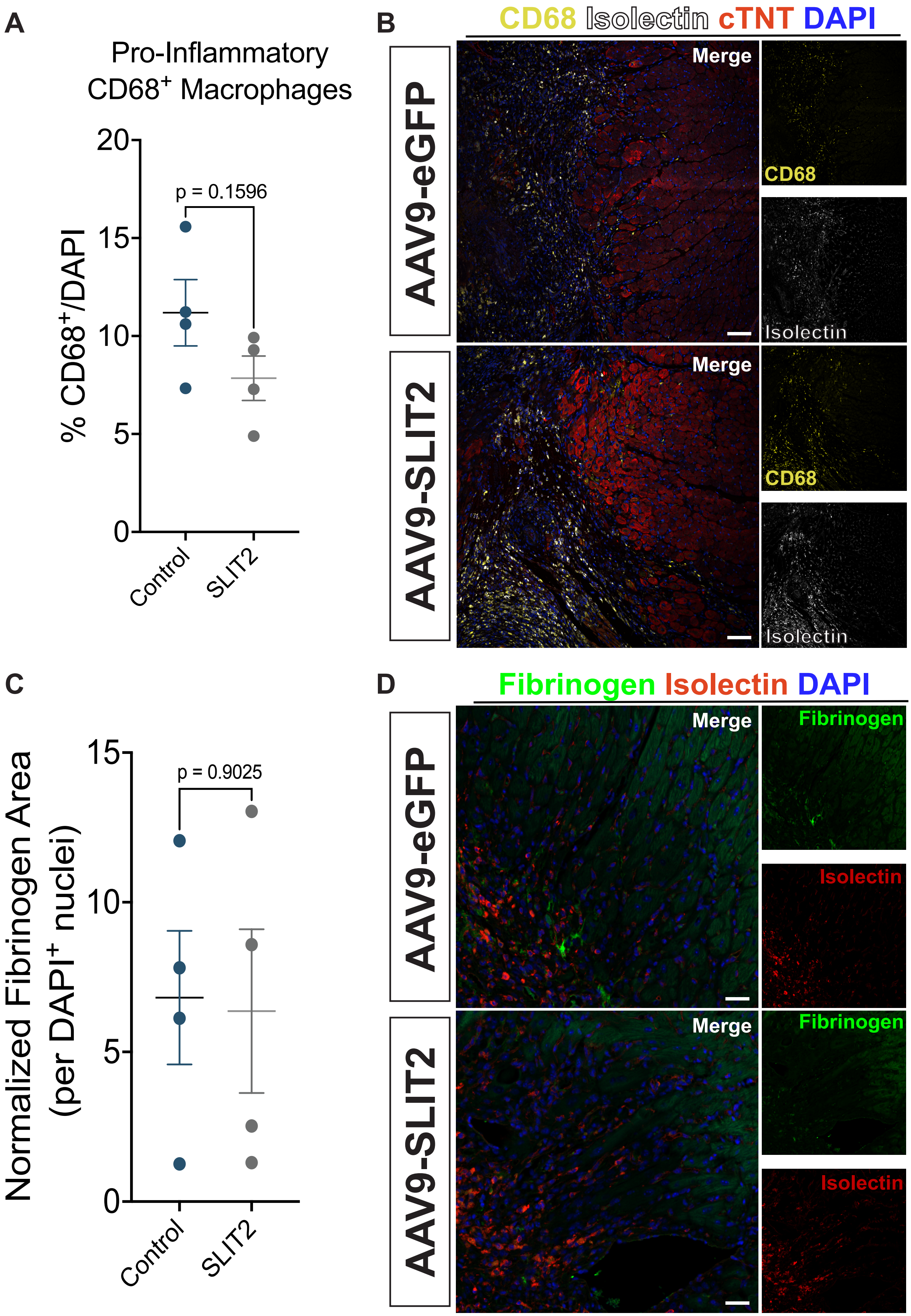

Supplemental Figure 6. Slit2 overexpression reduces cell death and macrophage infiltration 7 days post-MI. **(A)** Quantification of CD68+ pro-inflammatory macrophages (as % of total DAPI+ nuclei) in AAV9-eGFP or AAV9-Slit2-treated mice at 7 days post-MI. Data represent mean ± SEM; n=4 mice/group. **(B)** Representative immunofluorescence images of infarct border zone tissue showing CD68 (yellow), Isolectin B4 (white), cardiac troponin T (red), and DAPI (blue). **(C)** Quantification of fibrinogen deposition normalized to total DAPI nuclei in the infarct/border zone of AAV9-eGFP (control) and AAV9-Slit2-treated hearts. % Area of fibrinogen-positive signal was calculated from immunostained sections and divided by total DAPI nuclei per FOV, then scaled by 10^5. Data represent mean ± SEM; n=4 mice/group. **(D)** Representative immunofluorescence images of infarct border zone tissue stained for fibrinogen (green), isolectin β4 (red), and DAPI (blue). Scale bar = 20μm.

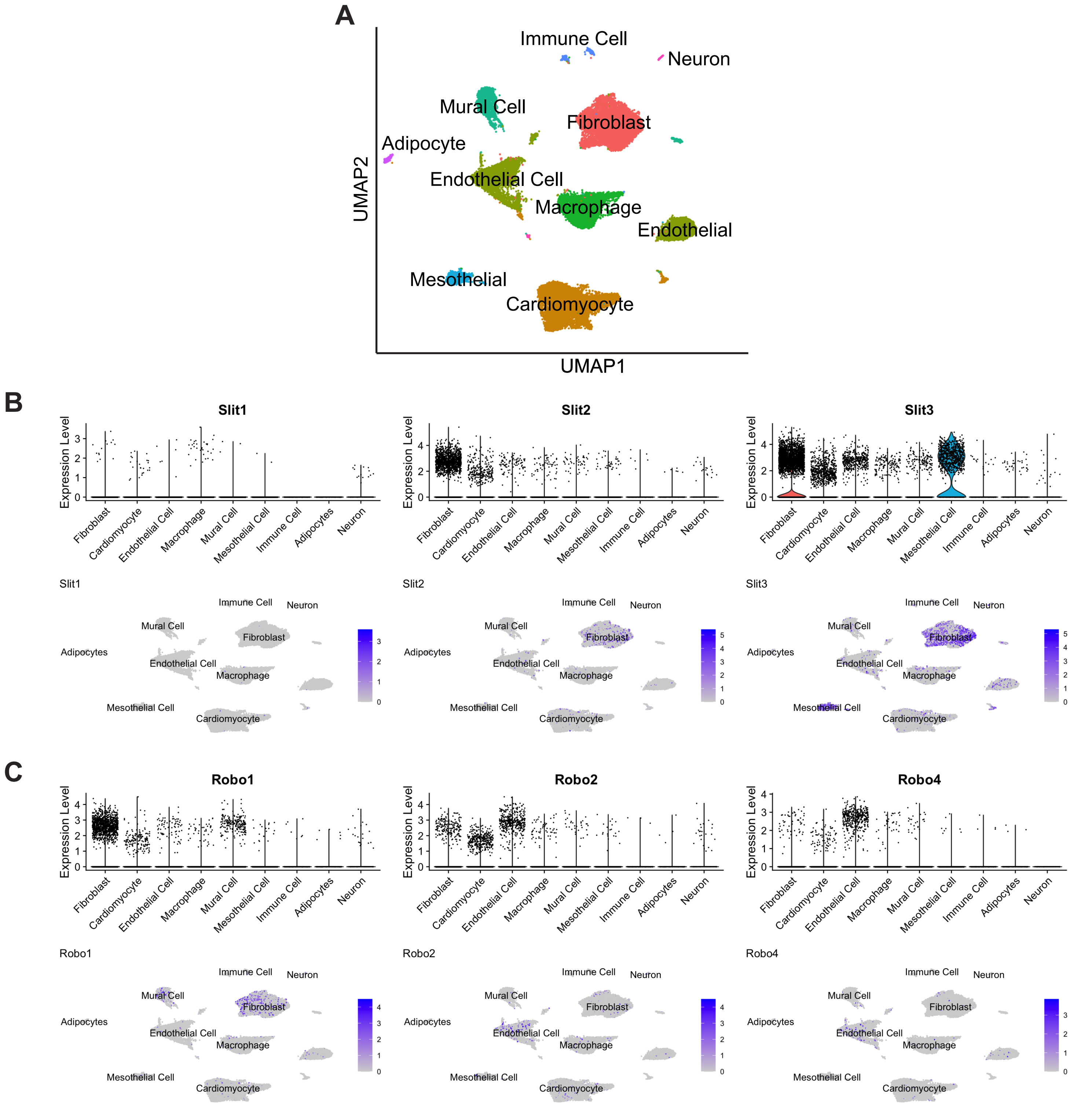

Supplemental Figure 7. Single nucleus RNA-sequencing reveals cell-type specific expression of Slit and Robo family members in both young and aged mouse hearts. (A) UMAP representation of cardiac cell clusters from integrated single-nucleus RNA-sequencing data from young (12-week-old) and aged (18-month-old) healthy male mouse hearts. A total of 27,808 nuclei were analyzed. Clusters were annotated according to their gene markers using Enrichr and PanglaoDB. **(B)** Violin plots showing the distribution and relative expression of *Slit1*, *Slit2*, and *Slit3* across cardiac cell types. *Slit2* is predominantly expressed in fibroblasts, while Slit3 shows enriched expression in fibroblasts, mesothelial cells, and adipocytes. *Slit1* expression is minimal across all clusters. **(C)** Expression of *Robo1*, *Robo2*, and *Robo4* across cell populations. *Robo1* is primarily expressed in fibroblasts and mural cells; *Robo2* is detected at low levels in neurons; *Robo4* is predominantly restricted to endothelial cells. Data sourced from a published dataset by Vidal et al., 2020.

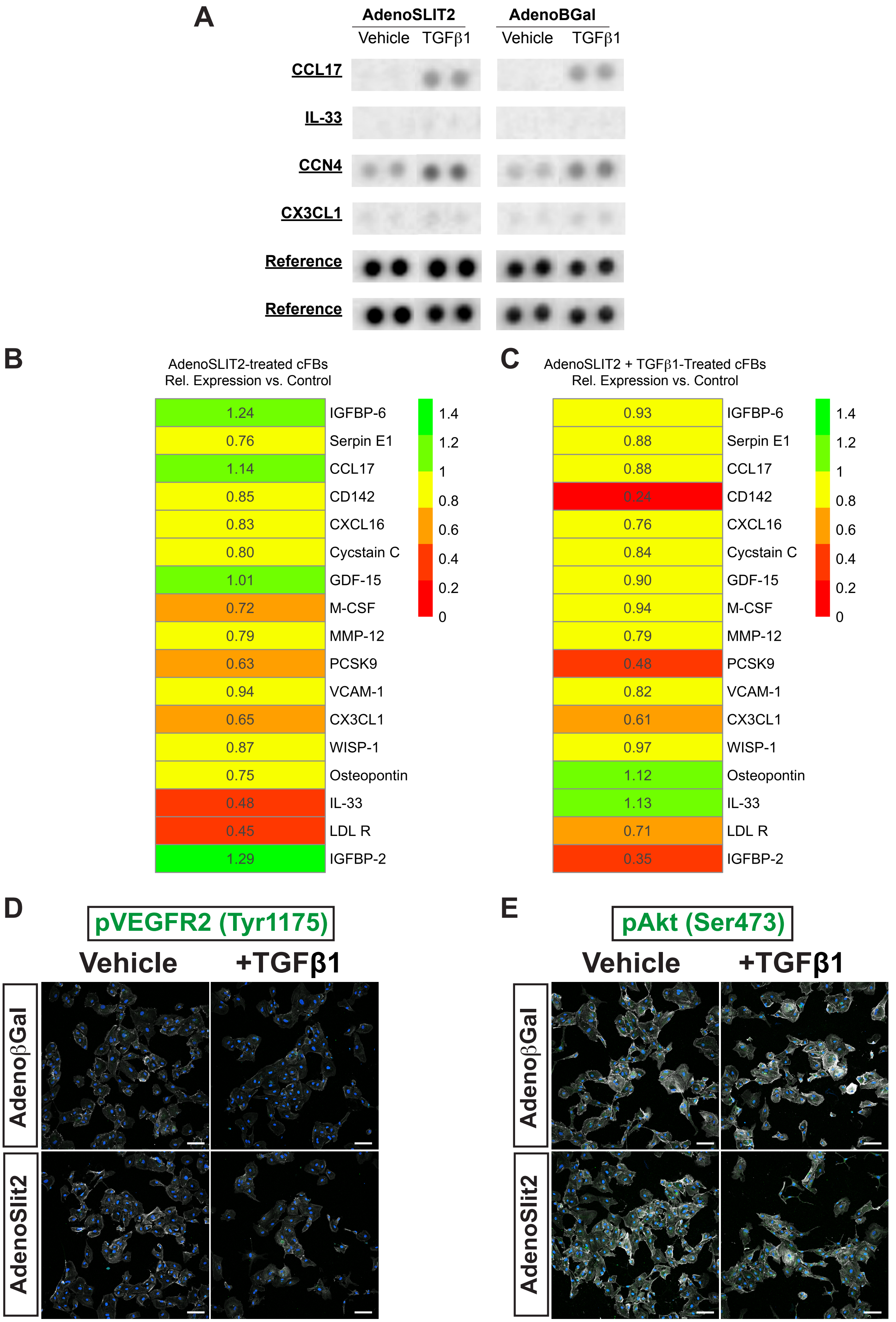

Supplemental Figure 8. Overexpression of Slit2 in cardiac fibroblasts alters the secretion of cytokines. **(A)** Representative cytokine antibody array blots of conditioned media from primary cardiac fibroblasts infected with AdenoSLIT2 or control AdenoβGal and treated with vehicle or TGFβ1. Spots corresponding to CCL17, IL-33, CCN4, and CX3CL1 are highlighted; two internal reference spots are shown for signal normalization. **(B)** Heatmap of fold-change in relative cytokine abundance in conditioned media from AdenoSLIT2-infected fibroblasts versus AdenoβGal controls under vehicle treatment. **(C)** Heatmap of fold-change in cytokine abundance in media from AdenoSLIT2-infected fibroblasts versues AdenoβGal controls following TGFβ1 treatment. Values in each cell denote the mean fold-change (SLIT2/βGal), and the color scale (0 to 1.4) reflects up- (green) or down-regulation (red) relative to control. **(D)** Immunohistochemical analyses of phosphorylated VEGFR2 (Tyr1175; green) and **(E)** phosphorylated AKT (Ser473; green) in HCAECs. Cells were co-labeled with phalloidan (white) and DAPI (blue). Scale bar, 20μm.
